# Diagnostic significance and carcinogenic mechanism of pan‐cancer gene POU5F1 in liver hepatocellular carcinoma

**DOI:** 10.1002/cam4.3486

**Published:** 2020-09-26

**Authors:** Dingdong He, Xiaokang Zhang, Jiancheng Tu

**Affiliations:** ^1^ Center for Gene Diagnosis, and Clinical Lab Zhongnan Hospital of Wuhan University Wuhan China

**Keywords:** biomarker, immune infiltrates, LIHC, pathogenesis, POU5F1

## Abstract

**Background:**

The prognostic and clinicopathological significance of POU Class 5 Homeobox 1 (POU5F1) among various cancers are disputable heretofore. The diagnostic value and functional mechanism of POU5F1 in liver hepatocellular carcinoma (LIHC) have not been studied thoroughly.

**Methods:**

An integrative strategy of meta‐analysis, bioinformatics, and wet‐lab approach was used to explore the diagnostic and prognostic significance of POU5F1 in various types of tumors, especially in LIHC. Meta‐analysis was utilized to investigate the impact of POU5F1 on prognosis and clinicopathological parameters in various cancers. The expression level and diagnostic value of POU5F1 were assessed by qPCR in plasma collected from LIHC patients and controls. The correlation between POU5F1 and tumor infiltrating immune cells (TIICs) in LIHC was evaluated by CIBERSORT. Gene set enrichment analysis (GSEA) was performed based on TCGA. Hub genes and related pathways were identified on the basis of co‐expression genes of POU5F1.

**Results:**

Elevated POU5F1 was associated with poor OS, DFS, RFS, and DSS in various cancers. POU5F1 was confirmed as an independent risk factor for LIHC and correlated with tumor occurrence, stage, and invasion depth. The combination of POU5F1 and AFP in plasma was with high diagnostic validity (AUC = 0.902, *p* < .001). Specifically, the level of POU5F1 was correlated with infiltrating levels of B cells, T cells, dendritic cells, and monocytes in LIHC. GSEA indicated that POU5F1 participated in multiple cancer‐related pathways and cell proliferation pathways. Moreover, CBX3, CCHCR1, and NFYC were filtered as the central hub genes of POU5F1.

**Conclusion:**

Our study identified POU5F1 as a pan‐cancer gene that could not only be a prognostic and diagnostic biomarker in various cancers, especially in LIHC, but functionally carcinogenic in LIHC.

## Graphical abstract

POU5F1 as a pan‐cancer gene could be a prognostic and diagnostic biomarker in various cancers, especially in LIHC. Elevated POU5F1 might promote the transformation of B cells naive into B cells memory in LIHC. POU5F1 may play a vital role in LIHC through CBX3, CCHCR1, and NFYC to regulate some cancer‐related pathways.

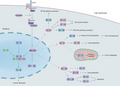

## 1 INTRODUCTION

Cancer has become a key influencing factor of morbidity and mortality in both developed and developing countries.^1^ There will be an escalating trend of death rates caused by cancers in the future due to deficient cognition in the pathological processes and regulatory mechanisms of cancers.^2^ Although the prognoses of cancers have been ameliorated through various therapeutic methods, the prognostic outcomes are invariably unsatisfactory in multiple kinds of cancers. Among all kinds of cancers, liver hepatocellular carcinoma (LIHC) plays one of the major roles. According to the cancer statistics data from 2020, LIHC ranks sixth in mortality among all cancers.^3^ As the main diagnostic and prognostic biomarker for LIHC, the sensitivity and specificity of α‐fetoprotein (AFP) were ungratified in the early diagnosis of LIHC. Consequently, urgent requirements are raised to find novel biomarkers as potential diagnostic indicators and therapeutic targets of LIHC.

Many studies have certified that cancer stem cells (CSCs) are associated with aggression, metastasis, and recrudescence in various cancers. In addition, several CSC markers have been proven to contribute to the poor prognosis of cancers,^4^, ^5^, ^6^, ^7^ indicating the significance of CSC markers in the prognosis of malignancies. However, due to the complexity of the regulatory network in tumor pathologic processes, the prognostic significance of CSC markers is not fully understood. With more in‐depth studies on CSC markers, some of these markers may become important targets in cancer diagnosis, therapy, and prognosis.

POU Class 5 Homeobox 1 (POU5F1) is a transcription factor of the POU family that binds an octameric sequence motif to activate the expression of downstream genes.^8^ POU5F1 has been identified as one of the most important CSC markers and participates in stemness maintenance in various tumors.^9^, ^10^ Published literatures have certified that increased POU5F1 was correlated with clinicopathological features and prognosis not only in LIHC, but also in bladder carcinoma, non‐small cell lung carcinoma, and oral squamous cell cancer.^11^, ^12^, ^13^, ^14^ POU5F1 may serve as an essential predictive factor for multiple cancers in the near future.

Although many studies have been performed, the prognostic significance of POU5F1 in cancers remains controversial, and the functions of POU5F1 in the regulatory network of tumors are not fully recognized. Some studies have come to different or even totally opposite conclusions regarding the prognostic value of POU5F1 and the role of POU5F1 in tumor development. For instance, He et al. showed that elevated POU5F1 in esophageal squamous cell carcinoma symbolized poor survival outcomes.^15^ However, Ge et al. found that high expression of POU5F1 was connected with longer survival in esophageal squamous cell carcinoma.^16^ The prognostic value of POU5F1 in LIHC was not statistically significant according to Qian et al^17^; but was prominent in studies performed by Huang et al.^18^ These disputes have not been settled in a reasonable way and the value of POU5F1 in tumor prognosis is still ambiguous. Current studies on the role of POU5F1 in LIHC mainly used tissue samples, hindering the clinical application of POU5F1 as a diagnostic biomarker due to its invasiveness. Studies focusing on the POU5F1 status in plasma could facilitate its promotion.

In this study, we adopted an integrative strategy of meta‐analysis, bioinformatics, and wet‐lab approach to explore the diagnostic and prognostic significance of POU5F1 in various types of tumors, especially in LIHC. First, we performed a meta‐analysis and trial sequential analysis (TSA) with a large sample size to evaluate the significance of POU5F1 for survival prognosis in various cancers. LIHC was selected as the major target when combining the meta‐analysis results with the survival analysis results from TCGA datasets. Then, we validated the POU5F1 expression level in plasma and evaluated the diagnostic value of POU5F1 in LIHC. Furthermore, a protein–protein interaction (PPI) network was constructed based on the co‐expression genes of POU5F1 and central hub genes were recognized. Finally, a cell signal transduction diagram was drawn to clarify the potential functional pathways of POU5F1 in LIHC.

## 2 MATERIALS AND METHODS

### 2.1 Literature search strategy

We comprehensively retrieved PubMed, Embase, Web of Science, and Cochrane Library to search studies published from 1 January 2000 to 1 June 2019 with language limitation in English and screened studies reporting prognosis and clinicopathological features in cancer patients with aberrant expression of POU5F1. To increase search sensitivity, we used a strategy involving both Medical Subject Heading terms and free‐text words. The search strategy was segmented into three parts: “POU5F1 transcription factor or octamer transcription factor 4 or octamer transcription factor 3”, “neoplasms or malignant neoplasms or carcinoma”, and “prognosis or prognostic factors or survival”. We also manually browsed the references of retrieved articles to recognize more eligible studies that might have been missed by the search strategy.

### 2.2 Literature inclusion and exclusion criteria

Published articles that met the seven criteria were enrolled: (a) evaluated the association between POU5F1 expression and clinical prognosis or clinicopathological parameters of cancers; (b) provided hazard ratios (HRs) and 95% CI or survival curves of POU5F1 relevant outcomes; (c) cohort studies (follow‐up duration longer than 24 months); (d) whole paper was written in English; (e) available full‐text articles; (f) research on humans; and (g) sample size of cancer patients was no less than 20. The exclusion criteria included the following: (a) absence of essential data, such as detection methods of POU5F1 expression, survival analysis data, and accurate prognosis indicators; and (b) reviews, case reports, letters, conference abstracts, animal trials, or duplicate publications.

### 2.3 Literature data extraction and quality evaluation

Each process of our research was strictly in conformity to preferred reporting items for systematic reviews and meta‐analyses (PRISMA) guidelines.^19^ Important features of the eligible cohorts were recorded, including first author; published year; nation; sample size; tumor category; age and gender of the patients; detection method and cut‐off value for POU5F1; follow‐up period; study design; clinicopathological parameters; outcome of interest, including overall survival (OS), disease‐free survival (DFS), disease‐specific survival (DSS), and recurrence‐free survival (RFS). The Newcastle–Ottawa Scale (NOS) was utilized to appraise the quality of the included cohorts. According to the NOS criteria, a cohort was considered of high quality when the total score was no less than 7.^20^

### 2.4 Trial sequential analysis

With new studies constantly enrolled in the cumulative meta‐analysis, type I and type II errors might increase due to repetitive tests of significance, fragmentary data, and ambiguous publication bias.^21^ Trial sequential analysis (TSA) can overcome these obstacles and estimate a priori information size (APIS), which is considered as the minimal sample size required to draw a reliable conclusion. When the cumulative Z‐curve fails to cross the conventional boundary (Z = 1.96), it indicates that the result is farfetched. If the Z‐curve crosses the conventional boundary but does not reach the TSA boundary, meaning the trials show false positive results. If the Z‐curve crosses both the conventional boundary and TSA boundary but not the APIS, it suggests that more researches are needed to support the conclusion. If the Z‐curve crosses all three boundaries, a reliable conclusion has been certified. We implemented TSA by maintaining two‐sided α of 5%, 15% relative risk reduction (RRR), and statistical test power of 80%. We performed TSA with a fixed‐effects model when I^2^ was less than 30%. Elsewise, random‐effects model would be executed.

### 2.5 Expression and survival analysis based on TCGA

The Tumor Immune Estimation Resource (TIMER) is an online database that incorporates expression profiles of 10,009 samples across 23 cancer types from TCGA (https://cistrome.shinyapps.io/timer/).^22^ We utilized TIMER to confirm the expression level of POU5F1 in various cancers. Gene Expression Profiling Interactive Analysis (GEPIA), another online analysis tool, contains survival and clinicopathological data extracted from various cancers based on TCGA (http://gepia.cancer‐pku.cn/).^23^ Survival analyses of OS and DFS were executed by GEPIA to find the correlation between POU5F1 expression and the prognosis of various cancers.

### 2.6 Specimens

Plasma specimens of 30 LIHC patients from Zhongnan Hospital of Wuhan Universitywere collected during July 2017 and October 2019 and stored at −80°C until use. LIHC patients were identified on the basis of their pathology reports. Meanwhile, 30 healthy people without hepatic diseases or abnormal liver biochemical outcomes were enrolled as controls. Our study was authorized by the Medical Ethics Committee of Zhongnan Hospital of Wuhan University.

### 2.7 RNA extraction and quantitative PCR analysis

RNA was extracted from plasma using Total RNA Separate Extraction Kit (Bioteke, China) according to the manufacturer's instructions. NanoDrop 2000C was applied to assess the concentration and purity of RNA. ReverTra Ace qPCR RT Kit (Toyobo, Japan) was used to reverse transcript mRNA into complementary DNA (cDNA). Quantitative PCR (qPCR) was implemented using SYBR Green I UltraSYBR Mixture (CWBIO) on Bio‐Rad CFX96 (Bio‐Rad Laboratories). *Glyceraldehyde 3*‐*phosphate dehydrogenase* (*GAPDH*) was used as the endogenous reference gene. The detailed sequences of each pair of primers are listed in Table S1. All experiments were repeated twice. The expression status of the target gene was assessed by the 2^−ΔCq^ method, in which ΔCq represents the value of the mean quantification cycle (Cq) of the target gene subtracting the mean Cq of the endogenous reference gene.

### 2.8 Tumor infiltrating immune cell reckoning

CIBERSORT provides a deconvolution algorithm that is able to distinguish 22 kinds of tumor infiltrating immune cells (TIICs) from other cell types in tissues.^24^ Expression profiles of 50 normal liver tissues and 374 LIHC tumor tissues were downloaded from TCGA database, and TIIC proportions of each sample were evaluated by R (version 3.6.2) on the basis of the CIBERSORT algorithm. Then, the TIIC proportions of normal liver tissues and LIHC tumor tissues were divided into two subgroups based on the median POU5F1 expression level and visualized through violin plots.

### 2.9 Gene set enrichment analysis

Gene set enrichment analysis (GSEA) is a bioinformatics method that inspects the statistical significance of a priori defined sets of genes and verifies the differences between two biological states.^25^ We divided TCGA LIHC samples into two phenotype subgroups on the grounds of the median expression level of POU5F1. Genes from the TCGA expression profiles were ranked in a list according to the degree of divergence between the high POU5F1 subgroup and the low POU5F1 subgroup through GSEA software 4.0. Then, Gene Ontology (GO) gene sets and Kyoto Encyclopedia of Genes and Genomes (KEGG) gene sets were analyzed to identify functional terms and pathways enriched in each phenotype subgroup. Gene set permutations were executed 1000 times for each analysis. The criteria of significantly enriched pathways were normalized *p* value < .05 and the absolute value of normalized enrichment score (NES) > 1.5.

### 2.10 Enrichment analysis of POU5F1 co‐expression genes

Co‐expression genes of POU5F1 were screened out by R based on the expression profiles of TCGA. The cut‐off line was set at *p* < .05, and the absolute value of Spearman correlation coefficient > 0.45. GO enrichment analysis and KEGG pathway analysis were performed using the R package “clusterProfiler”. *p* < .05 was taken as the statistically significant threshold.

### 2.11 PPI network establishment and hub gene identification

We utilized the Search Tool for the Retrieval of Interacting Genes (STRING) database to establish a protein–protein interaction (PPI) network and discover the relationship among co‐expression genes of POU5F1. The interaction score was set at 0.4 in the STRING database. Cytoscape was used to enhance the legibility of the PPI network on the basis of interaction data obtained from the STRING database. We considered genes that interacted directly with POU5F1 as central hub genes and those that directly interacted with the central hub genes as subordinate hub genes.

### 2.12 Statistical analysis

All statistical analyses in this study were performed using Stata SE15 (Stata Corporation), SPSS 25.0 (SPSS Inc.), GraphPad Prism 8.0 (GraphPad Inc.), and R (version 3.6.2). Amalgamative HRs and relating 95% CIs in the meta‐analysis were computed by Stata SE15. If the studies did not provide HRs or corresponding 95% CIs, these values were calculated by the equation: HR = (*P*0/(1 − *P*0))/(*P*1/(1 − *P*1)), in which P0 and P1 represented the survival rates of the decreased and elevated POU5F1 subgroups, respectively. The 95% CI was calculated through exp (lnHR ±1.96 × stderr), exp represented exponential, lnHR was natural logarithm of HR, and stderr meant standard error of HR. Several studies did not report the relevant data about survival rate in subgroups, and we utilized Engauge Digitizer Version 10.8 to collect representative data on Kaplan–Meier survival curves. Then, the extracted data were imported into a computation sheet obtained from Tierney et al. for estimation of HRs and 95% CIs.^26^ Heterogeneity of enrolled cohorts was evaluated through Chi‐square‐based Q and *I* ^2^ analyses. We ran a meta‐analysis with a fixed‐effects model when the heterogeneity was acceptable (*I* ^2^ < 50% or *p* > .05). Otherwise, the random‐effects model was performed. Sensitivity analysis was conducted by sequentially expurgating each cohort to evaluate the stability of the amalgamative result. Potential publication biases were detected through Begg's and Egger's analyses.

Continuous variables with normal distribution are described as the mean ±standard deviation (SD). Median and inter‐quartile ranges were used to describe abnormally distributed continuous variables. Student's *t* test and Mann‐Whitney *U* tests were utilized for comparisons between two groups. Correlation analyses were conducted by Pearson and Spearman correlation tests. Chi‐square test and Fisher's exact test were applied for evaluation of categorical variables. Cox proportional hazard regression was used for univariate and multivariate analyses. Odds ratios (ORs) were calculated by logistic regression. Receiver operating characteristic (ROC) curve was performed to assess the diagnostic values. The statistically significant threshold of the two‐sided *p* value was set at .05.

## 3 RESULTS

### 3.1 Literature search results and quality evaluation

The retrieval procedure is illustrated in Figure S1. The search of the databases obtained 1542 references. A total of 1205 articles remained after the exclusion of duplicates. About 909 records were excluded by scanning the titles and abstracts. 296 studies were evaluated by browsing the full‐text. Eventually, 57 studies containing 7401 patients were enrolled in our study.^10^, ^11^, ^12^, ^13^, ^14^, ^15^, ^16^, ^17^, ^18^, ^27^, ^28^, ^29^, ^30^, ^31^, ^32^, ^33^, ^34^, ^35^, ^36^, ^37^, ^38^, ^39^, ^40^, ^41^, ^42^, ^43^, ^44^, ^45^, ^46^, ^47^, ^48^, ^49^, ^50^, ^51^, ^52^, ^53^, ^54^, ^55^, ^56^, ^57^, ^58^, ^59^, ^60^, ^61^, ^62^, ^63^, ^64^, ^65^, ^66^, ^67^, ^68^, ^69^, ^70^, ^71^, ^72^, ^73^, ^74^ Sixteen types of cancers were included, including acute myeloid leukemia, bladder cancer, breast cancer, cervical cancer, colorectal cancer, esophageal cancer, gallbladder adenocarcinoma, gastric cancer, head and neck cancer, LIHC, lung cancer, neuroblastomas, ovarian cancer, pancreatic cancer, papillary renal cell carcinoma, and prostate cancer. The expression levels of POU5F1 were detected by immunohistochemistry (IHC) in 44 studies, qPCR in 10 studies, and immunofluorescence (IF) in the remaining three studies. The essential features of the enrolled studies are exhibited in Table S2. The quality assessments of the enrolled studies were implemented through the NOS, and 45 studies were rated as high‐quality studies with comprehensive scores greater than 7 points (Table S3).

### 3.2 Overall analysis of POU5F1 expression and cancer prognosis

**FIGURE 1:**
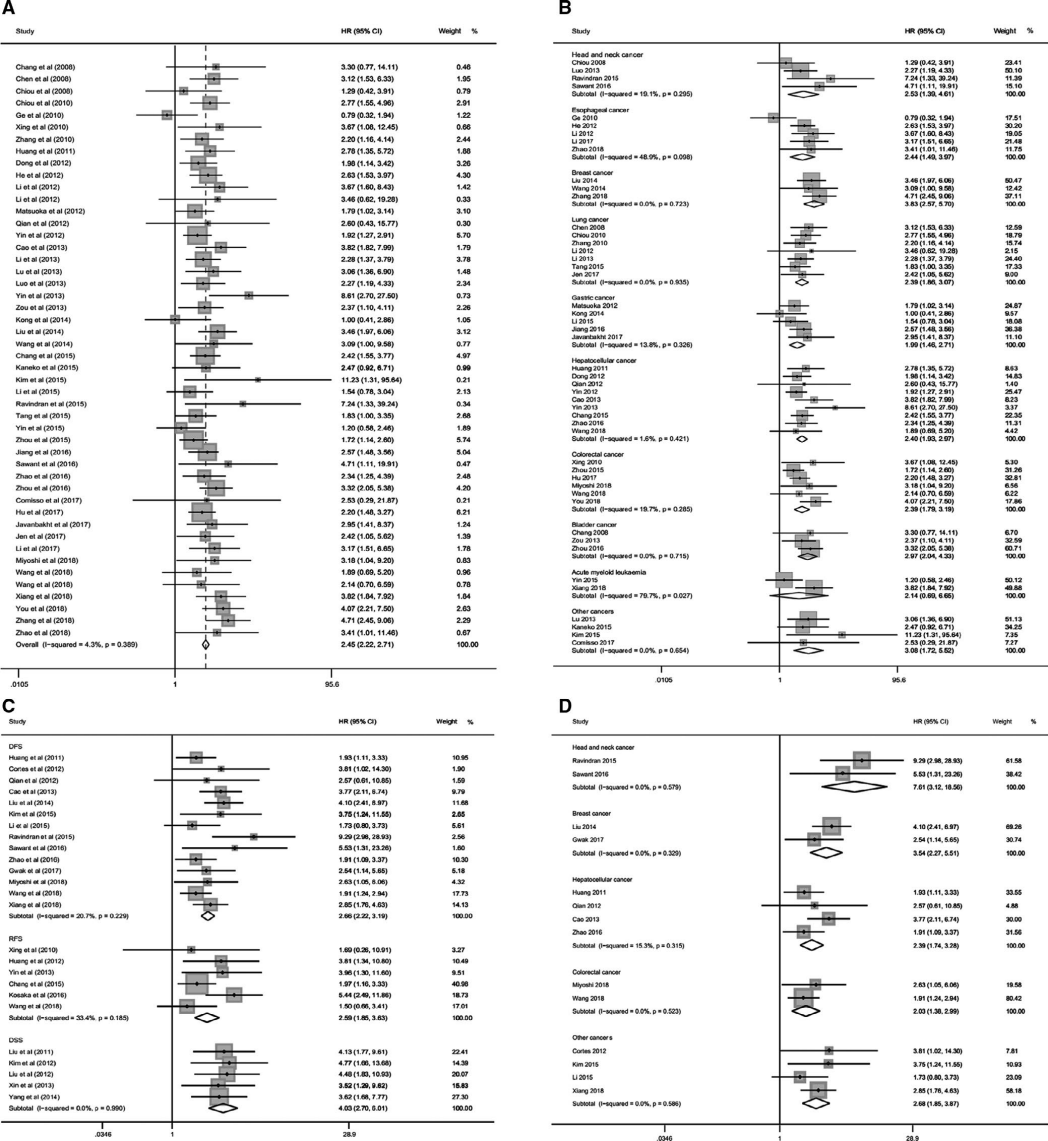
Forest plots of HRs for OS, DFS, RFS, and DSS with elevated POU5F1 expression. (A) HRs for OS. (B) HRs for OS subgroup analysis of cancer type. (C) HRs for DFS, RFS, and DSS. (D) HRs for DFS subgroup analysis of cancer type

Among the included 57 studies, a total of 5,485 subjects in 48 studies described the relationship between POU5F1 expression and OS, 1649 subjects in 14 studies for DFS, five studies with 1249 subjects for DSS and six studies involving 636 subjects for RFS. According to the meta‐analyses, the heterogeneities were not distinct in these four kinds of prognosis analyses (Figure 1A,C). Therefore, we capitalized on a fixed‐effects model to calculate the amalgamative HRs and relating 95% CIs. The results showed that increased POU5F1 was correlated with inferior outcomes for OS (HR = 2.45, 95% CI = 2.22−2.71, *p* < .001), DFS (HR = 2.66, 95% CI = 2.22−3.19, *p* < .001), DSS (HR = 4.03, 95% CI = 2.70−6.01, *p* < .001), and RFS (HR = 2.59, 95% CI = 1.85−3.63, *p* < .001).

### 3.3 Subgroup analysis for OS and DFS

Subgroup analyses were implemented for OS and DFS to clarify the connection between POU5F1 expression and cancer type, analysis type, sample size, and detection method. Studies were defined as “other cancers” in the cancer type subgroup when there was only one enrolled study for each kind of cancer. As demonstrated in Figure 1B and Table 1, elevated expression of POU5F1 predicted poor prognosis of OS in bladder cancer, breast cancer, colorectal cancer, esophageal cancer, gastric cancer, LIHC, head and neck cancer, lung cancer and other cancers, including ovarian cancer, cervical cancer, neuroblastomas, and pancreatic cancer. However, the prognostic value of POU5F1 was not obvious in the overall survival of acute myeloid leukemia patients. Simultaneously, overexpression of POU5F1 was related to shorter DFS in head and neck cancer, breast cancer, LIHC, colorectal cancer, and other cancers, including lung cancer, gastric cancer, cervical cancer, and acute myeloid leukemia (Figure 1D, Table S4). Furthermore, the subgroup category of analysis type, sample size, and detection method also indicated an observable relationship between a high level of POU5F1 and shorter OS and DFS.

**TABLE 1:** Subgroup analyses on pooled HRs of POU5F1 for OS

Categories		No. of studies	No. of patients	Pooled HR (95% CI)	Significant *z*	*p* value	Heterogeneity *I* ^2^ (%)	*p* value	Model
[1]	OS	48	5485	2.45 (2.22‐2.71)	17.74	.000	4.3	.389	Fixed
[2]	Cancer type								
1)	Head and neck cancer	4	321	2.44 (1.48‐4.02)	3.51	.000	19.1	.295	Fixed
2)	Esophageal cancer	5	450	2.49 (1.81‐3.45)	5.54	.000	48.9	.098	Fixed
3)	Breast cancer	3	343	3.83 (2.57‐5.70)	6.60	.000	0.0	.723	Fixed
4)	Lung cancer	7	706	2.39 (1.86‐3.07)	6.78	.000	0.0	.935	Fixed
5)	Gastric cancer	5	969	2.02 (1.53‐2.67)	4.93	.000	13.8	.326	Fixed
6)	Hepatocellular cancer	9	1082	2.39 (1.94‐2.95)	8.10	.000	1.6	.421	Fixed
7)	Colorectal cancer	6	752	2.31 (1.82‐2.94)	6.80	.000	19.7	.285	Fixed
8)	Bladder cancer	3	360	2.97 (2.04‐4.33)	5.67	.000	0.0	.715	Fixed
9)	Acute myeloid leukemia	2	239	2.14 (0.69‐6.66)	1.31	.190	79.7	.027	Random
10)	Other cancers	4	263	3.08 (1.72‐5.52)	3.79	.000	0.0	.654	Fixed
[3]	Analysis type								
1)	Multivariate	28	3405	2.61 (2.28‐2.99)	13.91	.000	1.6	.440	Fixed
2)	Univariate	20	2080	2.28 (1.97‐2.64)	11.10	.000	4.4	.402	Fixed
[4]	Sample size								
1)	≥110	22	3713	2.40 (2.12‐2.72)	13.83	.000	6.0	.380	Fixed
2)	<110	26	1772	2.54 (2.15‐2.99)	11.12	.000	5.6	.382	Fixed
[5]	Detection method								
1)	IHC	36	4197	2.35 (2.10‐2.63)	14.91	.000	0.0	.620	Fixed
2)	RT‐PCR	9	901	2.58 (1.99‐3.34)	7.21	.000	19.2	.272	Fixed
3)	IF	3	387	3.47 (2.41‐5.00)	6.68	.000	35.4	.213	Fixed

### 3.4 Correlation of POU5F1 and clinicopathological characteristics

To explore why elevated POU5F1 could lead to worse prognosis in various cancers, the correlations between POU5F1 status and neoplastic clinicopathological parameters were evaluated (Table 2). The overexpression of POU5F1 was remarkably correlated with tumor size, TNM stage, tumor differentiation, tumor invasion depth, lymph node metastasis, distant metastasis, lymphovascular invasion, vascular invasion, tumor number, and tumor recurrence. Non‐statistically significant results were found in age, gender, tumor encapsulation, liver cirrhosis, HBsAg, and smoking.

**TABLE 2:** Pooled ORs for the correlation between elevated POU5F1 and clinicopathological characteristics

Clinicopathological parameters	No. of studies	No. of patients	Risk of high POU5F1 OR (95% CI)	Significant *z*	*p* value	Heterogeneity *I* ^2^ (%)	*p* value	Model
Age (≥60 vs <60)	16	1694	1.08 (0.88‐1.32)	0.69	.489	0.0	.768	Fixed
Gender (Male vs Female)	35	3850	1.05 (0.90‐1.23)	0.65	.517	0.0	.844	Fixed
Tumor size (≥5 cm vs <5 cm)	14	1967	1.38 (1.13‐1.68)	3.21	.001	42.2	.048	Fixed
TNM stage (III‐IV vs I‐II)	22	2347	2.72 (2.23‐3.31)	9.99	.000	26.0	.130	Fixed
Tumor differentiation (Well‐Moderate vs Poor)	20	2632	3.08 (2.08‐4.56)	5.62	.000	67.0	.000	Random
Tumor invasion depth (T3–T4 vs T1–T2)	15	1861	2.31 (1.82‐2.93)	6.91	.000	10.6	.334	Fixed
Lymph node metastasis (Positive vs Negative)	25	3534	3.11 (2.66‐3.63)	14.31	.000	4.4	.400	Fixed
Distant metastasis (Positive vs Negative)	10	1437	2.86 (1.96‐4.19)	5.43	.000	0.0	.991	Fixed
Lymphovascular invasion (Positive vs Negative)	4	451	1.91 (1.25‐2.94)	2.96	.003	0.0	.640	Fixed
Vascular invasion (Positive vs Negative)	6	727	2.34 (1.65‐3.31)	4.80	.000	11.1	.345	Fixed
Tumor number (Multiple vs Single)	5	531	1.65 (1.06‐2.55)	2.23	.026	0.0	.812	Fixed
Tumor Recurrence (Positive vs Negative)	5	546	5.05 (3.33‐7.55)	7.62	.000	31.5	.212	Fixed
Tumor encapsulation (Incomplete vs Complete)	5	560	1.36 (0.95‐1.94)	1.69	.091	20.6	.283	Fixed
Liver cirrhosis (Positive vs Negative)	6	712	1.01 (0.68‐1.48)	0.03	.979	0.0	.817	Fixed
HBsAg (Positive vs Negative)	5	659	1.00 (0.62‐1.61)	0.01	.995	0.0	.793	Fixed
Smoke (Yes vs No)	4	406	1.30 (0.80‐2.10)	1.06	.287	0.0	.878	Fixed

### 3.5 Reliability of pooled prognostic results

TSA was implemented to assess the reliability of our meta‐analysis results (Figure S2). The heterogeneity of OS (I^2^ = 4.40%), DFS (I^2^ = 20.49%), and DSS (I^2^ = 0.00%) was not obvious, so the fixed model was utilized to perform TSA. However, heterogeneity appeared in RFS (I^2^ = 32.53%); thus, a random model was adopted. The accumulated Z‐curve of OS crossed the traditional boundary, TSA boundary, and APIS, suggesting that the conclusion was significantly reliable. The cumulative Z‐curves of DFS, DSS, and RFS crossed the conventional boundary and TSA boundary but did not reach the APIS, indicating that the current trials have obtained positive results, and more studies are required to support the results. Sensitivity analyses were executed to detect the stability of the conclusions about the prognostic value of POU5F1. No individual cohort could distinctly affect the pooled HRs of OS, DFS, DSS, or RFS, meaning the conclusions were credible (Figure S3). The underlying publication bias was appraised through Begg's and Egger's analyses. There was no potential publication bias found in OS, DFS, DSS, or RFS (Figure S4).

### 3.6 Expression and prognostic role of POU5F1 in various cancers

**FIGURE 2:**
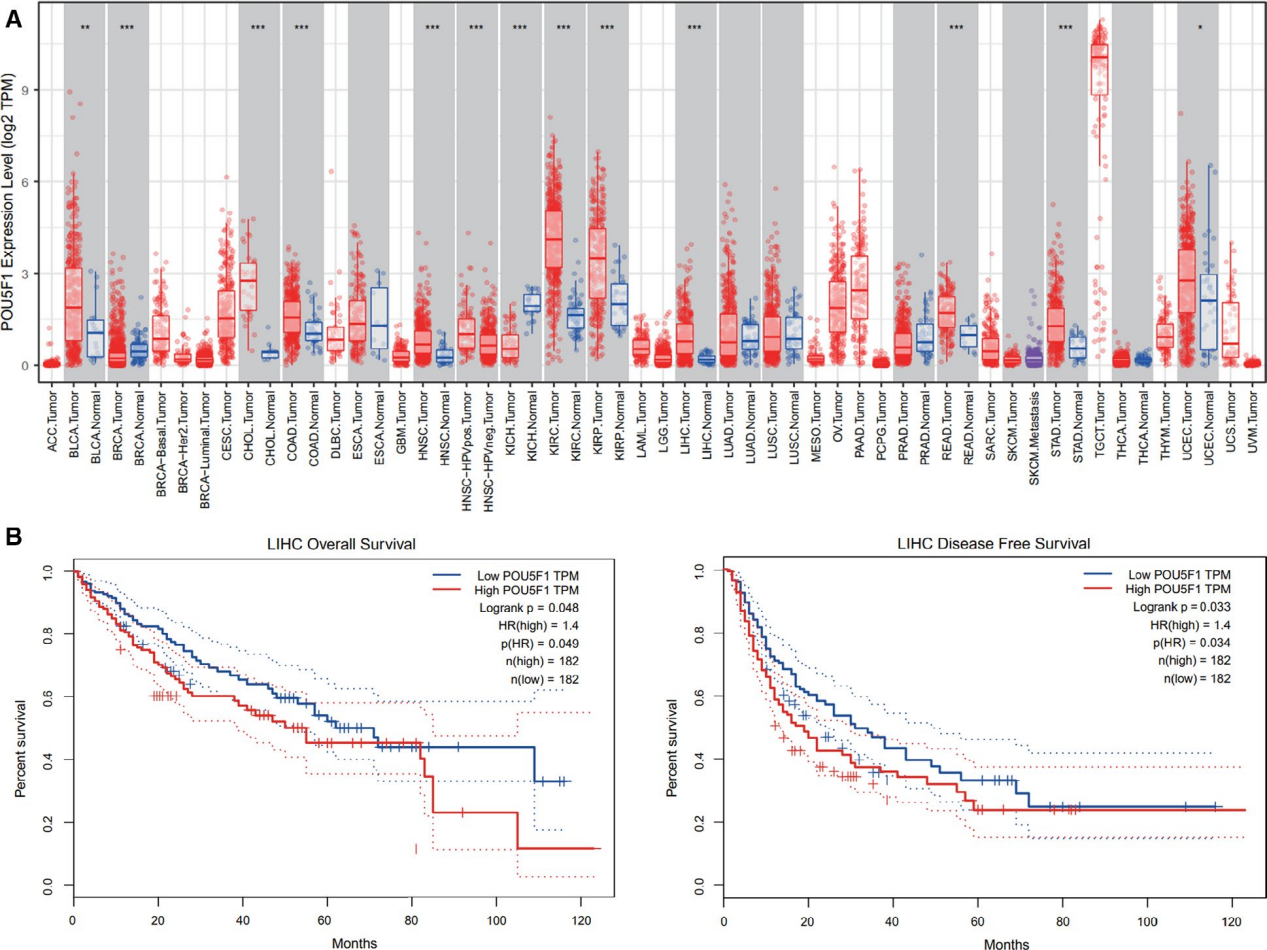
Expression status of POU5F1 in various cancers and survival analysis in LIHC. (A) Expression status of POU5F1 in various cancers based on TCGA. Statistical significance was assigned at *p* < .05 (*), *p* < .01 (**), *p* < .001 (***). (B) OS and DFS of LIHC patients with high (n = 182) or low (n = 182) POU5F1 levels in LIHC tissues

To further verify the expression level of POU5F1 in various cancers, TIMER was adopted to analyze the expression profiles from TCGA. As displayed in Figure 2A, POU5F1 was prominently upregulated in bladder urothelial carcinoma (BLCA), breast invasive carcinoma (BRCA), cholangiocarcinoma (CHOL), colon adenocarcinoma (COAD), head and neck squamous cell carcinoma (HNSC), kidney renal clear cell carcinoma (KIRC), kidney renal papillary cell carcinoma (KIRP), LIHC, rectum adenocarcinoma (READ), stomach adenocarcinoma (STAD), and uterine corpus endometrial carcinoma (UCEC). Interestingly, downregulation of POU5F1 was only observed in kidney chromophobe (KICH). Furthermore, survival analyses were carried out through GEPIA based on TCGA. Among the above‐mentioned cancers, only LIHC showed statistically significant differences in both OS and DFS (Figure 2B, Figure S5). Hence, LIHC was selected as the main target to explore the underlying functional role of POU5F1.

### 3.7 Association between POU5F1 and clinicopathological variables of LIHC

**FIGURE 3:**
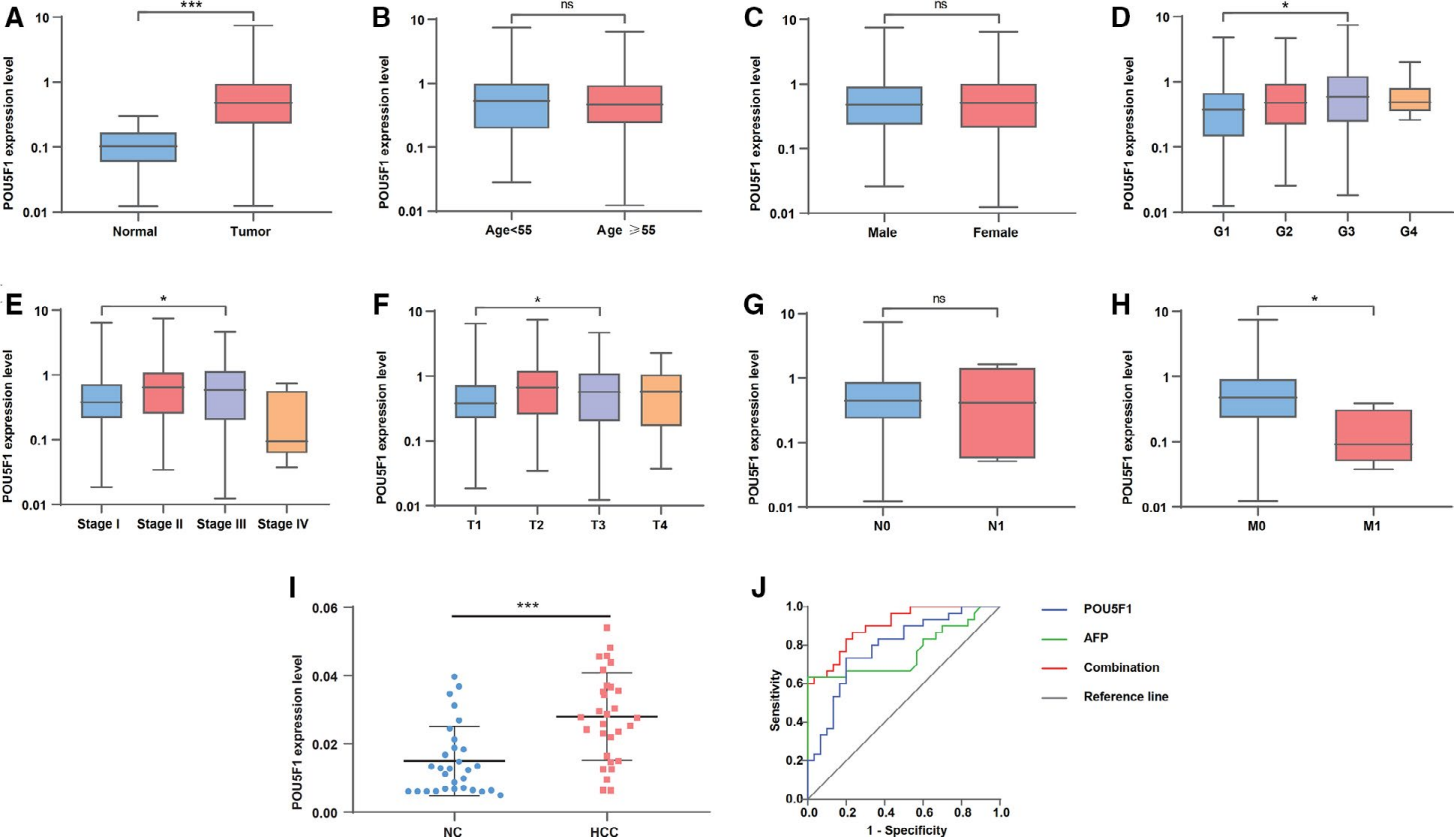
Association between POU5F1 expression and clinicopathologic characteristics and the diagnostic value of POU5F1 in LIHC. (A) Cancer status. (B) Age. (C) Gender. (D) Grade. (E) Clinical stage. (F) Tumor invasion depth. (G) Lymph node metastasis. (H) Distant metastasis. (I) Expression level of POU5F1 in plasma collected from 30 controls and 30 LIHC patients. (J) ROC based on POU5F1 and AFP levels in plasma separately or combinedly

The expression profiles and clinical characteristics of 374 LIHC patients were obtained from TCGA to probe the relationship between POU5F1 expression status and clinicopathological characteristics. As shown in Figure 3A‐H, elevated POU5F1 was associated with tumor occurrence (*p* < .001), advanced histological grade (*p* = .016), stage (*p* = .025), tumor invasion depth (*p* = .019), and distant metastasis (*p* = .018). Logistic regression analysis indicated the expression of POU5F1 as a risk factor that was associated with poor prognostic clinicopathologic variables (Table 3). Increased POU5F1 was significantly correlated with tumor occurrence (OR = 65.63, *p* < .001), advanced stage (OR = 2.06, *p* = .007), and tumor invasion depth (OR = 2.00, *p* = .001). In addition, POU5F1 (HR = 1.64, *p* = .038) was identified as an independent risk factor for OS of LIHC through multivariate analysis, as were tumor stage (HR =1.51, *p* < .001), tumor invasion depth (HR = 1.51, *p* < .001), and distant metastasis (HR = 3.73, *p* = .026) (Table 4).

**TABLE 3:** Correlations between elevated POU5F1 and clinicopathological characteristics in LIHC patients based on TCGA

Clinical characteristics	Total (N)	Risk of high POU5F1 OR (95% CI)	*p* value
Status (Tumor free vs With tumor)	421	65.63 (8.97‐480.14)	<.001
Age	370	1.00 (0.98‐1.01)	.820
Gender (Male vs Female)	371	1.07 (0.69‐1.65)	.767
Grade (III vs I)	177	1.89 (0.99‐3.61)	.054
Stage (III vs I)	256	2.06 (1.22‐3.50)	.007
T (III vs I)	261	2.00 (1.17‐3.41)	.011
N (Positive vs Negative)	256	1.10 (0.15‐7.93)	.925
M (Positive vs Negative)	270	0.26 (0.03‐2.32)	.225

**TABLE 4:** Univariate and multivariate analysis of OS in LIHC patients based on TCGA

Clinicopathologic variable	Univariate analysis	*p* value	Multivariate analysis	*p* value
	HR (95% CI)		HR (95% CI)	
Age	1.01 (1.00‐1.03)	.064		
Gender (Male vs Female)	1.18 (0.82‐1.69)	.380		
Grade (III vs I)	1.09 (0.82‐1.44)	.560		
Stage (III vs I)	1.63 (1.31‐2.02)	.000	1.51 (1.20‐1.89)	<.001
T (III vs I)	1.61 (1.30‐1.99)	.000	1.51 (1.21‐1.88)	<.001
N (Positive vs Negative)	1.94 (0.48‐7.93)	.355		
M (Positive vs Negative)	3.88 (1.22‐12.35)	.022	3.73 (1.17‐11.87)	.026
POU5F1 (High vs Low)	1.92 (1.34‐2.76)	.000	1.64 (1.03‐2.62)	.038

### 3.8 Diagnostic value of POU5F1 in plasma

We detected the expression level of POU5F1 in plasma collected from 30 LIHC patients and 30 normal controls by qPCR to investigate the diagnostic value of POU5F1. The main clinical characteristics of the enrolled subjects are listed in Table 5. Significantly higher alanine aminotransferase (ALT) (*p* < .001), aspartate aminotransferase (AST) (*p* < .001), γ‐glutamyl transferase (GGT) (*p* = .047), AFP (*p* < .001), and glucose (GLU) (*p* < .001) levels were observed in LIHC patients. In contrast, albumin (ALB) was much lower in LIHC patients than in normal controls (*p* < .001). The qPCR results revealed that POU5F1 was upregulated in the plasma of LIHC patients, which was consistent with the results from liver tissue samples based on TCGA (Figure 3I). Moreover, elevated POU5F1 was associated with a high level of ALT in plasma (*p* < .001) (Table 6). ROC analysis was utilized to assess the diagnostic value of POU5F1 in LIHC. As displayed in Figure 3J, the predictive validity of POU5F1 (AUC = 0.790, Se = 73.3%, Sp =80.0%, *p* < .001) was higher than that of AFP (AUC = 0.766, Se = 63.3%, Sp = 100.0%, *p* < .001). Encouragingly, the diagnostic validity was remarkably improved through the combination of POU5F1 and AFP (AUC = 0.902, Se = 83.3%, Sp = 80.0%, *p* < .001).

**TABLE 5:** The main clinical features of research subjects

Characteristics	Control (n = 30)	LIHC (n = 30)	*p* value
Gender			.007
Male (%)	17 (56.67)	27 (90.00)	
Female (%)	13 (43.33)	3 (10.00)	
Age (y)			.070
<55 (%)	19 (63.33)	11 (36.67)	
≥55 (%)	11 (36.67)	19 (63.33)	
ALT (U/L)	18.00 (13.00‐24.00)	43.00 (23.50‐68.00)	<.001
AST (U/L)	21.50 (18.00‐27.00)	49.50 (31.25‐83.00)	<.001
ALP (U/L)	88.00 (73.25‐157.00)	98.00 (78.50‐220.00)	.414
GGT (U/L)	24.50 (18.75‐47.25)	34.50 (23.75‐71.50)	.047
TP (g/L)	69.20 (60.60‐72.73)	63.05 (59.10‐72.80)	.232
ALB (g/L)	44.95 (42.83‐46.75)	35.90 (33.08‐38.50)	<.001
CEA (ng/mL)	1.88 (1.23‐2.55)	2.10 (1.50‐3.11)	.179
AFP (ng/mL)	2.77 (1.72‐3.65)	34.83 (2.47‐311.20)	<.001
GLU (mmol/L)	5.08 (4.42‐5.33)	5.86 (5.02‐7.49)	<.001

**TABLE 6:** Relationship between POU5F1 expression and clinical characteristics of LIHC patients

Characteristics	Patient number (n = 30)	Low expression (n = 15)	High expression (n = 15)	*p* value
Gender				.999
Male	27	13 (48.15)	14 (51.85)	
Female	3	2 (66.67)	1 (33.33)	
Age				.450
<55	11	4 (36.36)	7 (63.64)	
≥55	19	11 (57.89)	8 (42.11)	
AFP (ng/mL)				.450
<200	19	11 (57.89)	8 (42.11)	
≥200	11	4 (36.36)	7 (63.64)	
CEA (µg/L)				.999
<5	27	13 (48.15)	14 (51.85)	
≥5	3	2 (66.67)	1 (33.33)	
ALT (u/L)				<.001
<46	18	14 (77.78)	4 (22.22)	
≥46	12	1 (8.33)	11 (91.67)	
AST (u/L)				.715
<46	14	8 (57.14)	6 (42.86)	
≥46	16	7 (43.75)	9 (56.25)	
GGT (u/L)				.700
<55	20	9 (45.00)	11 (55.00)	
≥55	10	6 (60.00)	4 (40.00)	

### 3.9 Relationship between POU5F1 and TIICs

**FIGURE 4:**
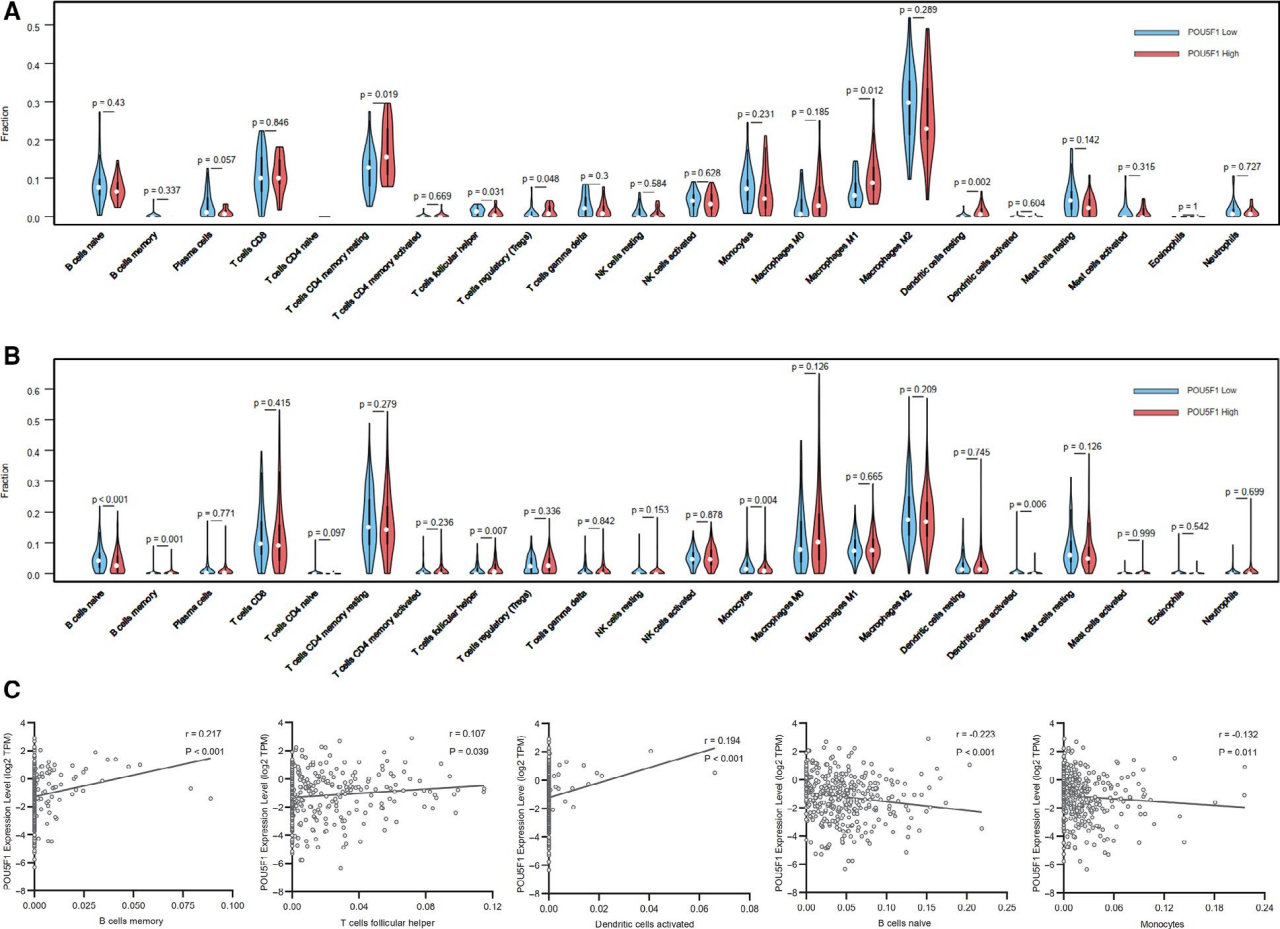
The proportion of 22 subpopulations of TIICs in normal liver tissues and LIHC tissues. (A) TIICs in normal liver tissues. (B) TIICs in LIHC tissues. Correlation between POU5F1 level and (C) B cells memory, (D) T cells follicular helper, (E) dendritic cells activated, (F) B cells naive, and (G) monocytes

To inquire into the mechanism of POU5F1 involved in the pathological progression of LIHC, we analyzed the correlation between POU5F1 expression and 22 types of TIICs through the CIBERSORT algorithm on the basis of expression profiles from TCGA. As exhibited in Figure 4A, T cells CD4 memory resting (*p* = .019), T cells regulatory (*p* = .048), macrophage M1 (*p* = .012), and dendritic cells resting (*p* = .002) increased in the high POU5F1 group of normal liver tissues, while T cells follicular helper (*p* = .031) decreased. In LIHC tumor tissues, B cells memory (*p* = .001) and T cells follicular helper (*p* = 007) were enriched in the high POU5F1 group, and B cells naive (*p* < .001), monocytes (*p* = .004), and dendritic cells activated (*p* = .006) were increased in the low POU5F1 group (Figure 4B). In addition, B cells memory (*p* < .001), T cells follicular helper (*p* = .039), and dendritic cells activated (*p* < .001) were positively related to POU5F1 in LIHC tumor tissues (Figure 4C‐E). The anomalous correlation between POU5F1 and dendritic cells activated might be partially explained by the limited data from dendritic cells activated. Negative correlations were observed in B cells naive (*p* < .001) and monocytes (*p* = .011) with POU5F1 (Figure 4F, G).

### 3.10 Identification of POU5F1‐related pathways

**FIGURE 5:**
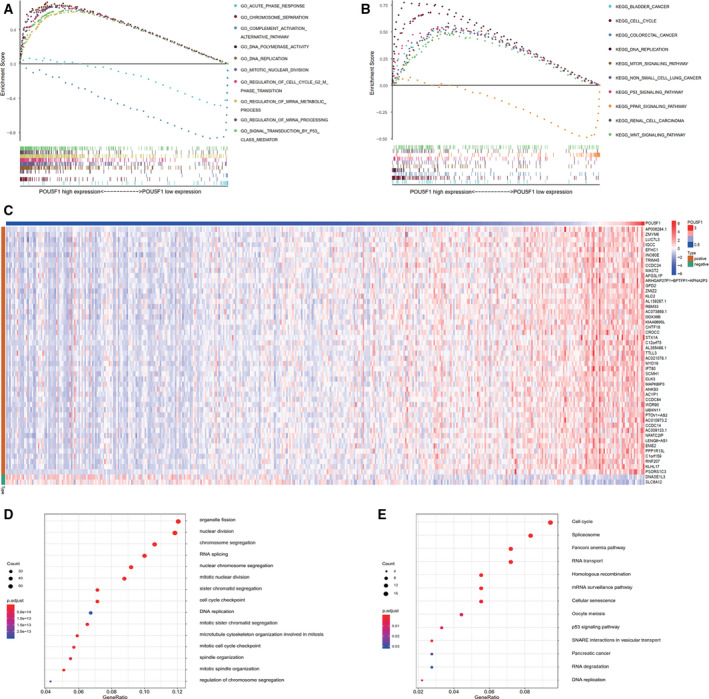
GSEA for POU5F1 and enrichment analysis of the co‐expression genes of POU5F1 in LIHC. (A) GSEA of POU5F1 based on GO gene sets. (B) GSEA of POU5F1 based on KEGG gene sets. (C) Representative expression heat map of the top 50 co‐expression genes of POU5F1. (D) GO enrichment analysis of the co‐expression genes of POU5F1. (E) KEGG enrichment analysis of the co‐expression genes of POU5F1

POU5F1‐related signaling pathways were analyzed through GSEA to identify pathways that were differentially activated in LIHC between low and high POU5F1 expression phenotypes. GO terms enriched in the high POU5F1 phenotype mainly contained DNA replication, regulation of cell cycle G2M phase transition, signal transduction by p53 class mediator and so on. GO terms, including acute phase response and complement activation alternative pathway, were enriched in the low POU5F1 phenotype (Figure 5A). Multiple cancer‐related KEGG pathways were enriched in the high POU5F1 phenotype, such as bladder cancer, colorectal cancer, non‐small cell lung cancer, and renal cell carcinoma. Several well‐known cancer‐related signaling pathways were also enriched in the high POU5F1 phenotype, including the MTOR signaling pathway, p53 signaling pathway, and WNT signaling pathway. The PPAR signaling pathway was enriched in the low POU5F1 phenotype (Figure 5B).

### 3.11 Enrichment analysis of POU5F1 co‐expression genes

To explore genes that might potentially be associated with POU5F1, co‐expression analysis was performed, and the expression status of the top 50 genes are displayed in Figure 5C. GO functional enrichment analysis indicated that these genes were enriched in cell proliferation‐related terms, including chromosome segregation, nuclear division, organelle fission, and DNA replication (Figure 5D). Cell cycle, spliceosome, p53 signaling pathway, pancreatic cancer, and DNA replication were the main signaling pathways in which these POU5F1 co‐expression genes were enriched through KEGG pathway analysis (Figure 5E).

### 3.12 PPI network construction and hub gene recognition

**FIGURE 6:**
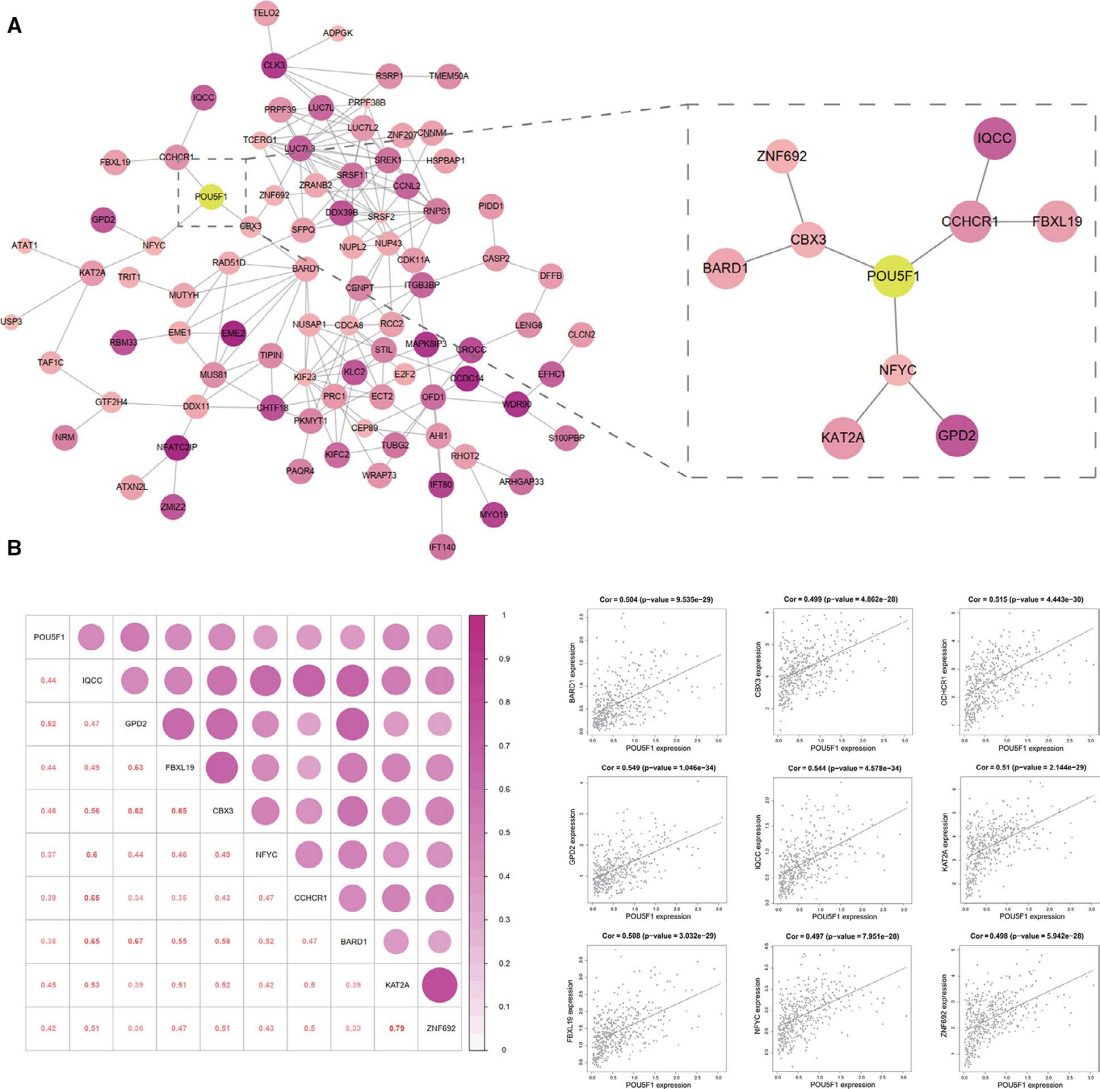
PPI network and the correlation between POU5F1 and the hub genes. (A) PPI network and the nine hub genes interacted with POU5F1. (B) Correlation between expression of POU5F1 and the nine hub gene

**FIGURE 7:**
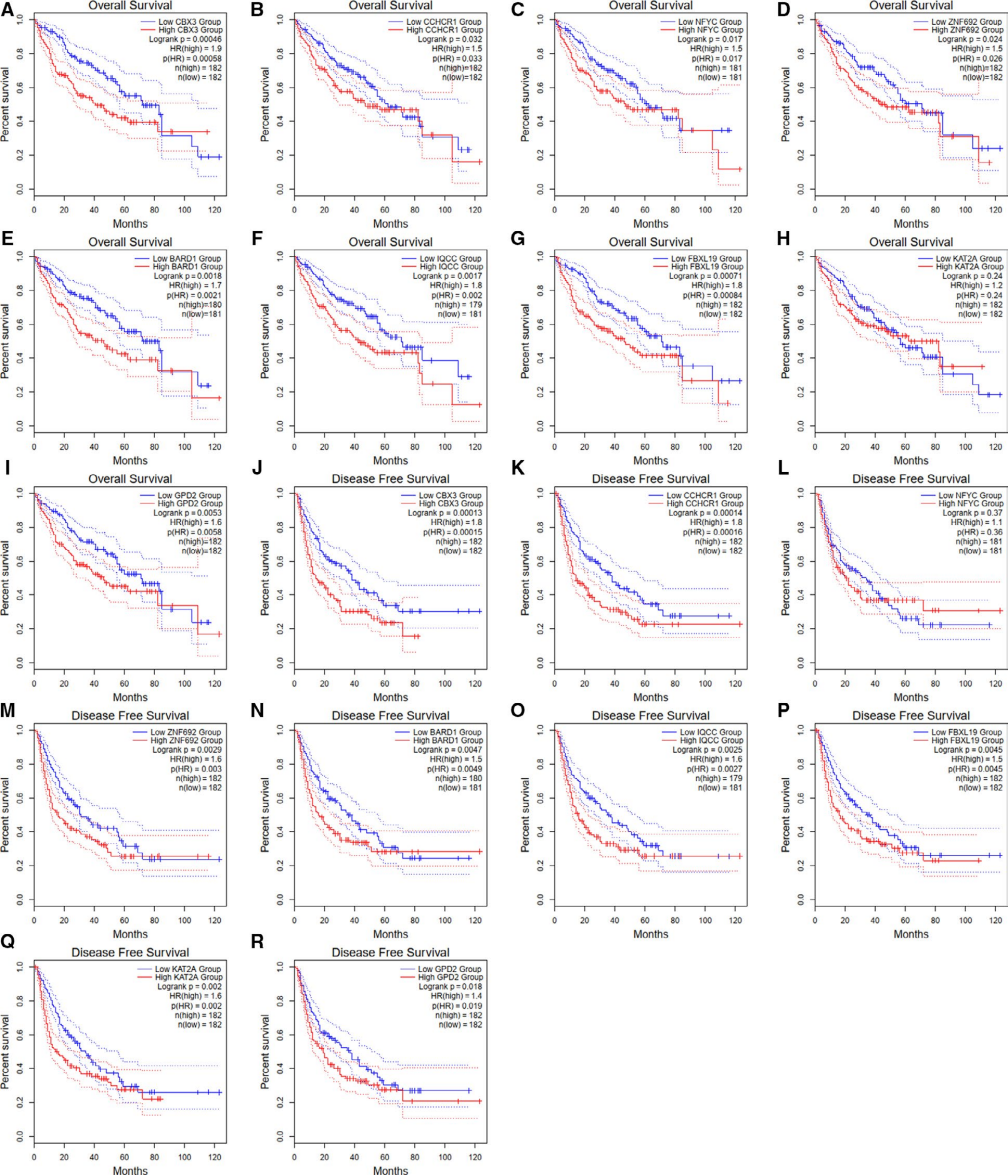
Kaplan–Meier survival analysis of nine hub genes of POU5F1 in LIHC based on TCGA. (A‐I) Overall survival of nine hub genes. (J‐R) Disease‐free survival of nine hub genes

**FIGURE 8:**
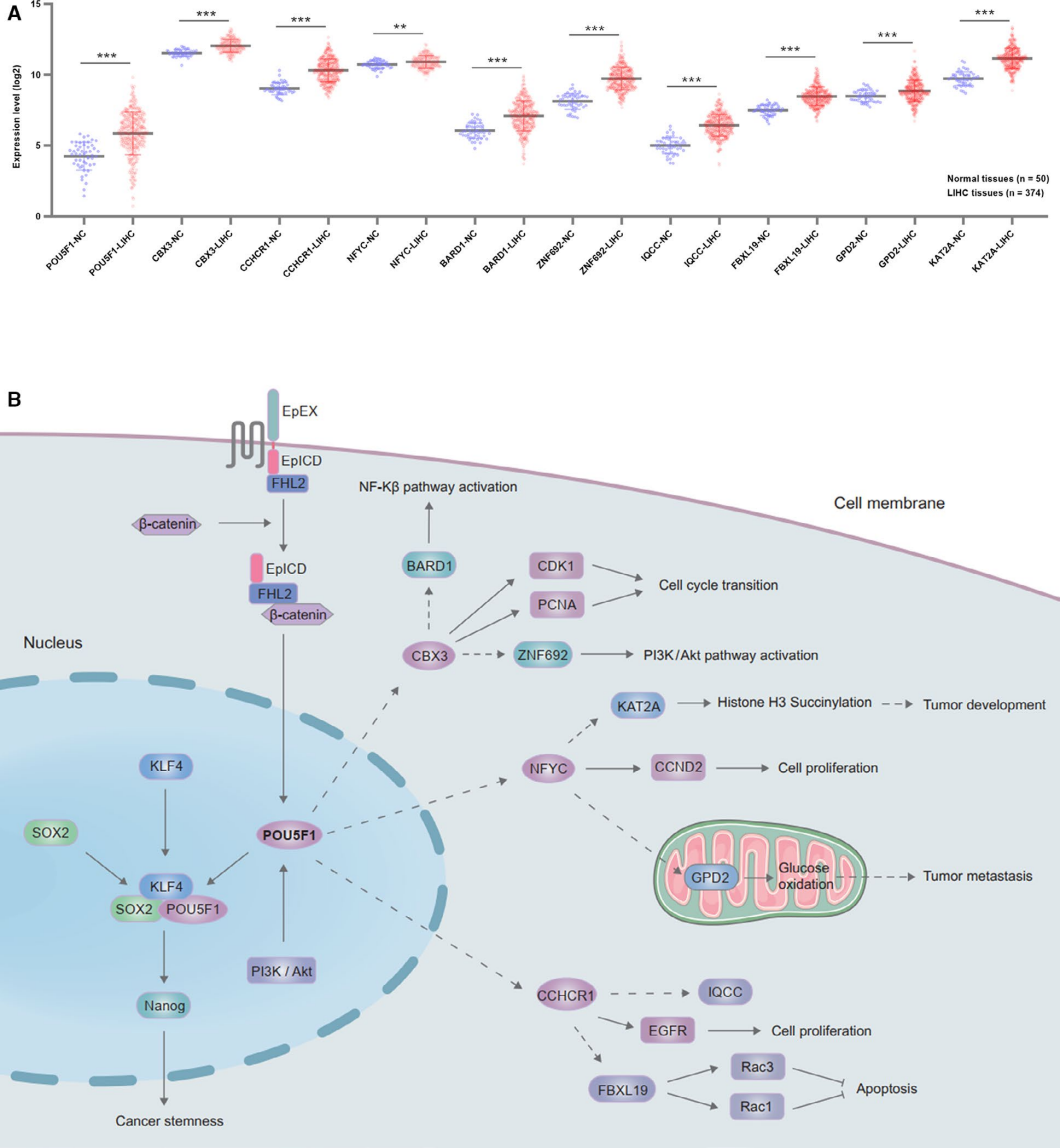
Expression levels and potential cell signal transduction pathways of POU5F1 and hub genes in LIHC. (A) Expression levels of POU5F1 and the nine hub genes in LIHC. (B) Potential cell signal transduction pathways of POU5F1 and the nine hub genes in LIHC

A PPI network was constructed to reveal the intrinsic correlations among the POU5F1 co‐expression genes. As exhibited in Figure 6A, the deeper color of each gene circle indicated an increased correlation coefficient with POU5F1. Analogously, a larger circle size indicated a smaller *P* value. Three genes (CBX3, CCHCR1, and NFYC) were found to be directly associated with POU5F1 and were defined as central hub genes. BARD1, ZNF692, IQCC, FBXL19, GPD2, and KAT2A had direct connections with the central hub genes and were regarded as subordinate hub genes for POU5F1. The expression status of the central hub genes and subordinate hub genes were all positively correlated with the expression level of POU5F1 in LIHC on the basis of TCGA (Figure 6B). In addition to KAT2A, shorter OS of LIHC was found to be correlated with the overexpression of all the hub genes (Figure 7A‐I). Elevated expression of all the hub genes except NFYC indicated poor DFS of LIHC (Figure 7J‐R). In addition, the nine hub genes were all prominently upregulated in LIHC patients based on TCGA (Figure 8A).

## 4 DISCUSSION

POU5F1 has been studied for a long period of time as a well‐known CSC marker that participates in tumor invasion, differentiation, and recurrence.^67^ A growing number of studies have suggested the prognostic value of POU5F1 in various malignancies. However, due to the limitation of sample size and methodology, the conclusions drawn by individual studies may be unauthentic to demonstrate the prognostic validity of POU5F1. We performed a meta‐analysis that incorporated 16 types of cancers with 7401 subjects from 57 studies to come to more reliable conclusions. The amalgamative results indicated that elevated POU5F1 was associated with poor OS, DFS, DSS, and RFS in various cancers. In particular, TSA confirmed that the sample size of current studies has far exceeded the APIS, suggesting it was quite credible to draw a conclusion that elevated POU5F1 was apparently connected with shorter OS in various cancers. Besides, the pooled estimates of clinicopathological parameters suggested that POU5F1 played pivotal roles in tumorigenesis, tumor growth, invasion, metastasis, and therapy resistance in multiple cancers. These results indicated that POU5F1 might serve as a prognostic pan‐cancer biomarker and potential therapeutic target.

POU5F1 was upregulated in BLCA, BRCA, CHOL, COAD, HNSC, KIRP, LIHC, READ, and STAD based on TCGA, which was consistent with the meta‐analysis results. Interestingly, differences in both OS and DFS between the high POU5F1 group and the low POU5F1 group were observed only in LIHC on the basis of TCGA, indicating that POU5F1 played a unique and important role in the prognosis of LIHC. Furthermore, DNA replication, regulation of cell cycle G2M phase transition, bladder cancer, colorectal cancer, non‐small cell lung cancer, renal cell carcinoma, MTOR signaling pathway, p53 signaling pathway, and WNT signaling pathway were the main GO and KEGG terms enriched in the high POU5F1 phenotype according to the GSEA. The GSEA results suggested that POU5F1 might participate in the pathological progression of LIHC and other cancers by promoting cell proliferation. Similar GO terms and KEGG pathways were found in the co‐expression genes of POU5F1 and further validated the GSEA results.

Previous studies reported that TIICs could independently predict OS among cancer patients and reflect the status of lymph nodes.^75^ Our study found that there was a prominent decrease in B cells naive and an increase in B cells memory in the high POU5F1 group of LIHC tumor tissues, hinting that the elevated POU5F1 might promote the transformation of B cells naive into B cells memory in LIHC. T cells follicular helper decreased in the high POU5F1 group in normal liver tissues, but increased in the high POU5F1 group in LIHC tumor tissues. The exact opposite results were found in dendritic cells activated. The typing and quantity conversion of TIICs in normal tissues and tumor tissues indicated the significant meanings of POU5F1 in regulating the tumor immune microenvironment of LIHC. The mechanism by which POU5F1 participates in the regulation of the tumor immune microenvironment still needs further study.

We identified upregulated POU5F1 as an independent prognostic factor for poor prognosis of LIHC through Cox regression, along with tumor stage, invasion depth, and distant metastasis. Overexpression of POU5F1 was related to high levels of ALT in plasma. Considering that a high concentration of ALT was indicative of liver cell destruction, we speculated that the overexpression of POU5F1 might be associated with hepatocellular necrosis or apoptosis.^76^ In addition, although the diagnostic value of POU5F1 in LIHC was quite gratifying, the necessity of applying POU5F1 and AFP together in the diagnosis of LIHC to improve the diagnostic specificity needed to be emphasized, in view of POU5F1, was upregulated in a variety of cancers and might reduce the diagnostic specificity.

The molecular regulation mechanisms and pathways by which POU5F1 participates in LIHC have not been thoroughly studied. To further explore the role of POU5F1 in LIHC, we constructed a PPI network using co‐expression genes of POU5F1 and identified hub genes that interacted with POU5F1, including CBX3, CCHCR1, NFYC, BARD1, ZNF692, IQCC, FBXL19, GPD2, and KAT2A. Based on related studies of hub genes, we visualized the pathways POU5F1 might play a role in LIHC (Figure 8B). It has been reported that the transcription factor complex of POU5F1, SOX2, and KLF4 binds to the Nanog promoter to induce cellular reprogramming and cancer stemness.^77^ EpICD translocates to the nucleus in a multiprotein complex and enhances the expression of POU5F1 by binding to the promoter of POU5F1.^78^ CBX3 has been confirmed to promote cell cycle transition by inducing CDK1 and PCNA.^79^ The elevated POU5F1 in LIHC may influence the expression of CBX3 and then activate the NF‐Kβ and PI3K/Akt pathways through BARD1 and ZNF692.^80^, ^81^ The promotion effect of CCND2 on cell proliferation is regulated by NFYC and may also be affected by POU5F1.^82^ The interaction between NFYC and KAT2A indicates that POU5F1 participates in tumor development through KAT2A‐mediated histone H3 succinylation.^83^ GPD2 promotes HuH‐7 cell mitochondrial energy metabolism which may be regulated by NFYC and POU5F1.^84^ As a central hub gene of POU5F1, CCHCR1 accelerates cell proliferation through EGFR.^85^ FBXL19 induces Rac1 and Rac3 expression and inhibits apoptosis.^86^ The relationship among FBXL19, CCHCR1, and POU5F1 needs further verification. In addition, loss of function of POU5F1 remarkably restrains propagation, metastasis, and aggression of cancer stem cells through inhibition of the PI3K/Akt pathway, from which we could expect POU5F1 to be an underlying target for cancer therapy.^87^

## 5 CONCLUSION

In summary, our study identified POU5F1 as a pan‐cancer gene with significant prognostic value in various cancers, especially in LIHC. POU5F1 can serve as an independent prognostic factor for LIHC, and the combination of AFP and POU5F1 in plasma has prominent diagnostic validity for LIHC. POU5F1 may influence the progression of LIHC by regulating the tumor immune microenvironment and participating in cell proliferation‐related pathways. Further research should be performed to verify the functional mechanism of POU5F1 in the pathogenesis of LIHC.

## CONFLICT OF INTEREST

The authors declare that they have no competing interests.

## AUTHOR CONTRIBUTIONS

JCT conceived and designed the workflow. DDH and XKZ performed the experiments and analyzed the data. DDH and XKZ wrote the manuscript. JCT revised the manuscript. All authors approved the final manuscript.

## ETHICS APPROVAL AND CONSENT TO PARTICIPATE

All experimental schemes were approved by the Ethics Committee of Zhongnan Hospital of Wuhan University.

## CONSENT FOR PUBLICATION

Not applicable.

## Supporting information

Fig S1

Fig S2

Fig S3

Fig S4

Fig S5

Table S1

Table S2

Table S3

Table S4

## Data Availability

The datasets used and/or analyzed during the current study are available from the corresponding author on reasonable request.
