# Supplementary figures and images for: Diagnostic significance and carcinogenic mechanism of pan‐cancer gene POU5F1 in liver hepatocellular carcinoma
Source: Cancer Med. 2020 Sep 26;9(23):8782–800. doi: 10.1002/cam4.3486 (PMC7724499; doi:10.1002/cam4.3486)

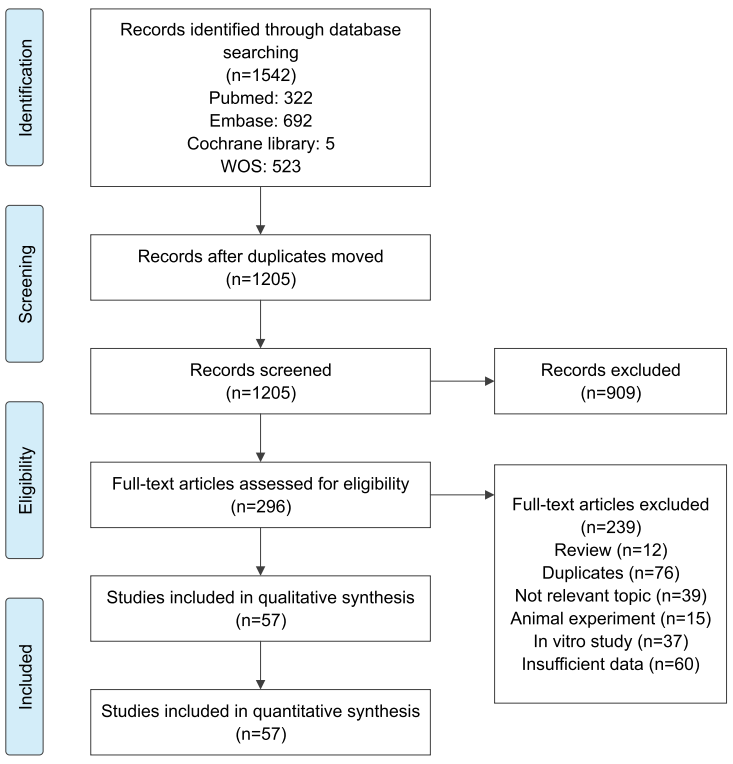


**Supplementary Figure S1.** Flow diagram of study selection process.

Supplement: Supplementary file 1 — Fig S1 [file CAM4-9-8782-s001.docx]

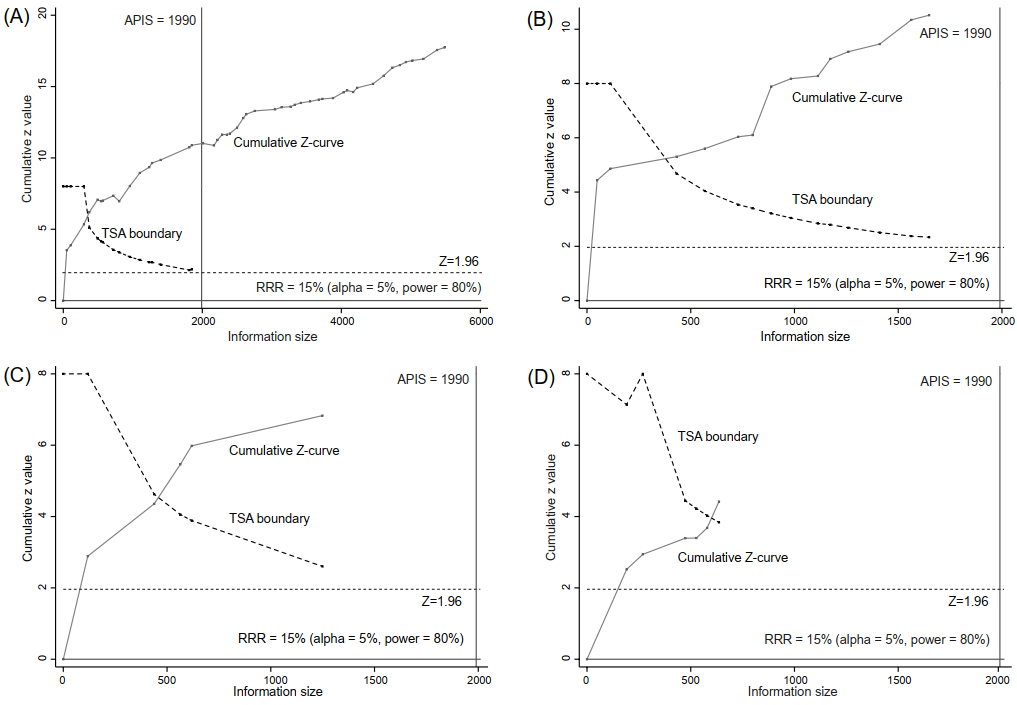


**Supplementary Figure S2.** TSA for OS, DFS, DSS and RFS based on APIS. (A) OS; (B) DFS; (C) DSS; (D) RFS.

Supplement: Supplementary file 2 — Fig S2 [file CAM4-9-8782-s002.docx]

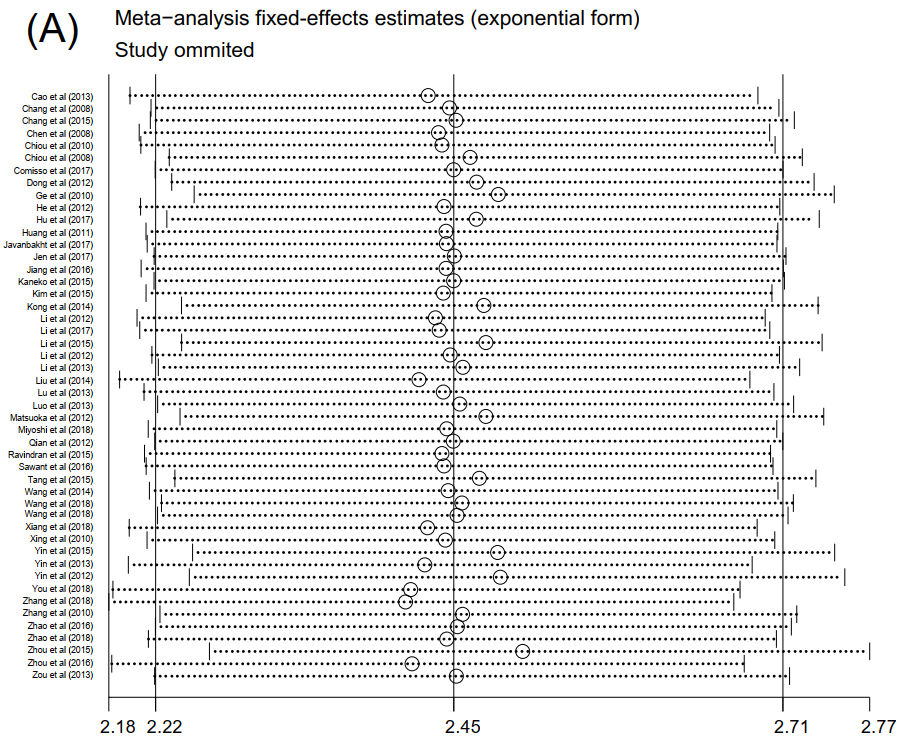


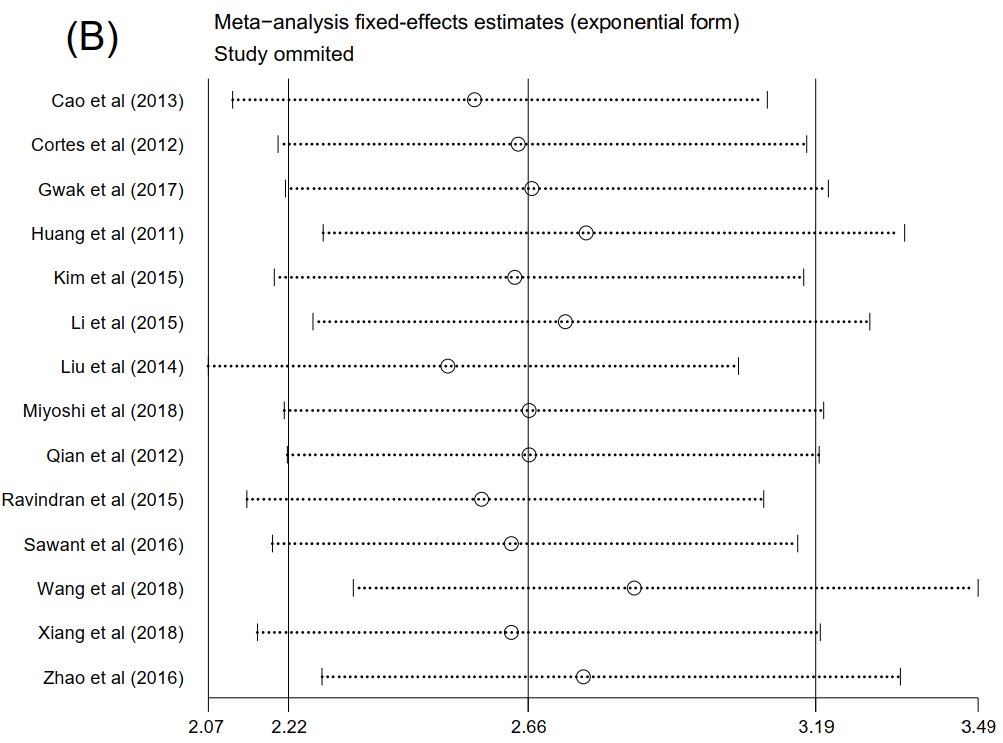


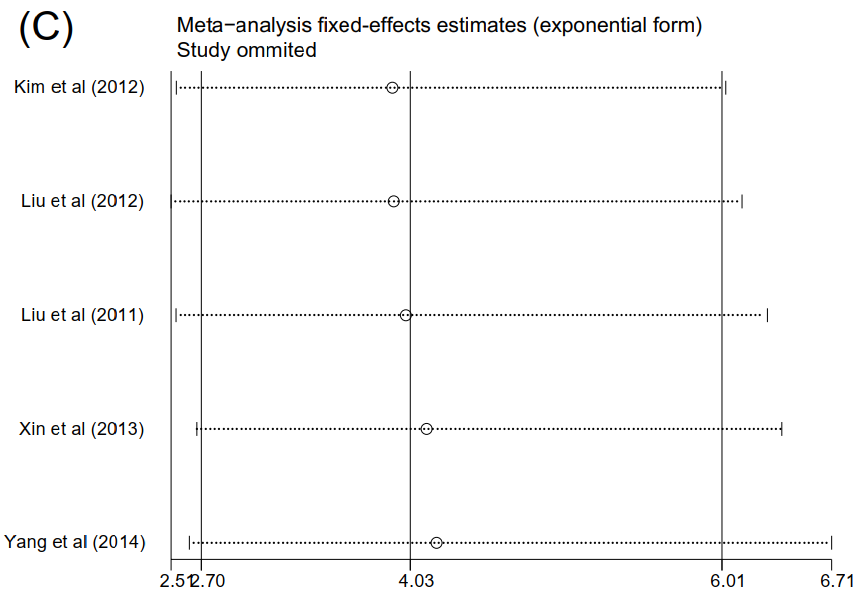


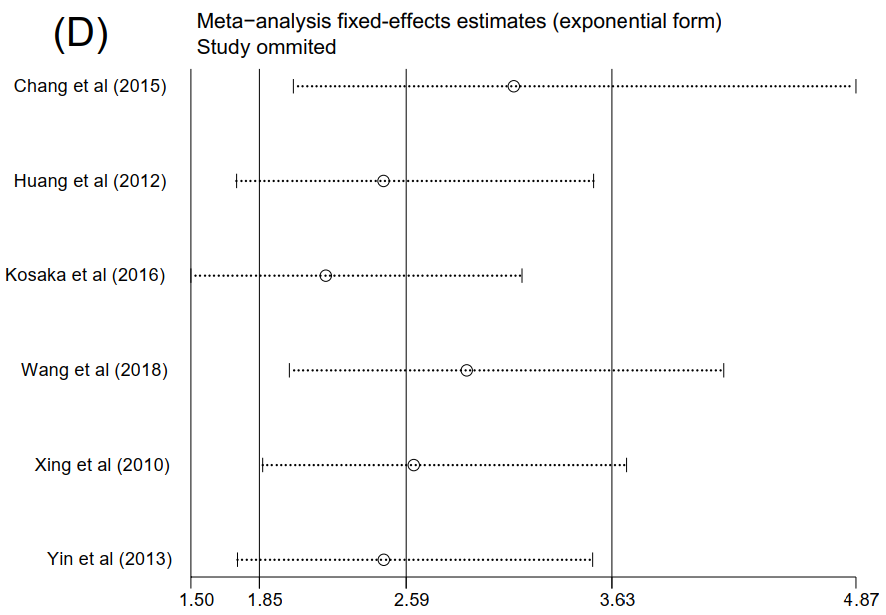


**Supplementary Figure S3.** Sensitivity analysis of prognostic value of POU5F1. (A) OS; (B) DFS; (C) DSS; (D) RFS.

Supplement: Supplementary file 3 — Fig S3 [file CAM4-9-8782-s003.docx]
