# Supplementary material for: Diagnostic significance and carcinogenic mechanism of pan‐cancer gene POU5F1 in liver hepatocellular carcinoma
Source: Cancer Med. 2020 Sep 26;9(23):8782–800. doi: 10.1002/cam4.3486 (PMC7724499; doi:10.1002/cam4.3486)
Supplement: Supplementary file 4 — Fig S4 [file CAM4-9-8782-s004.docx]

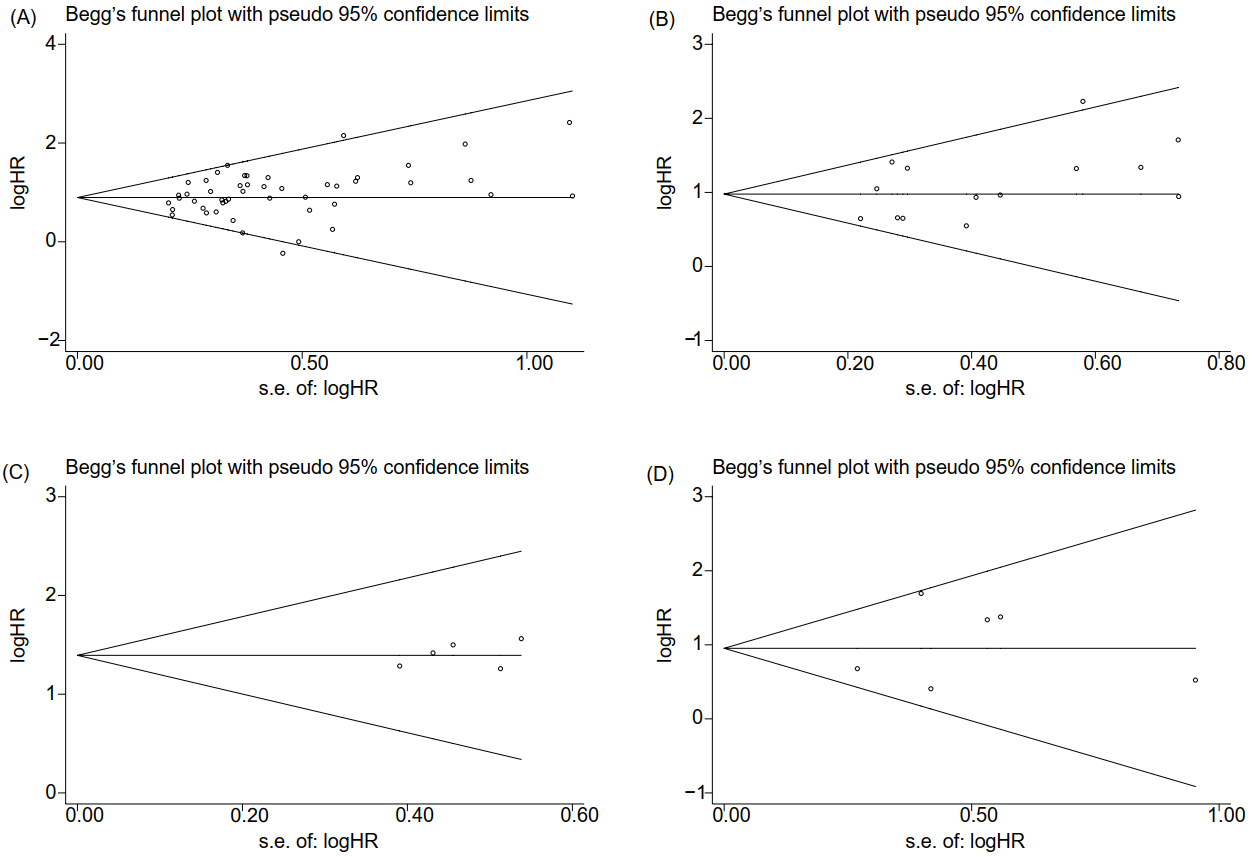

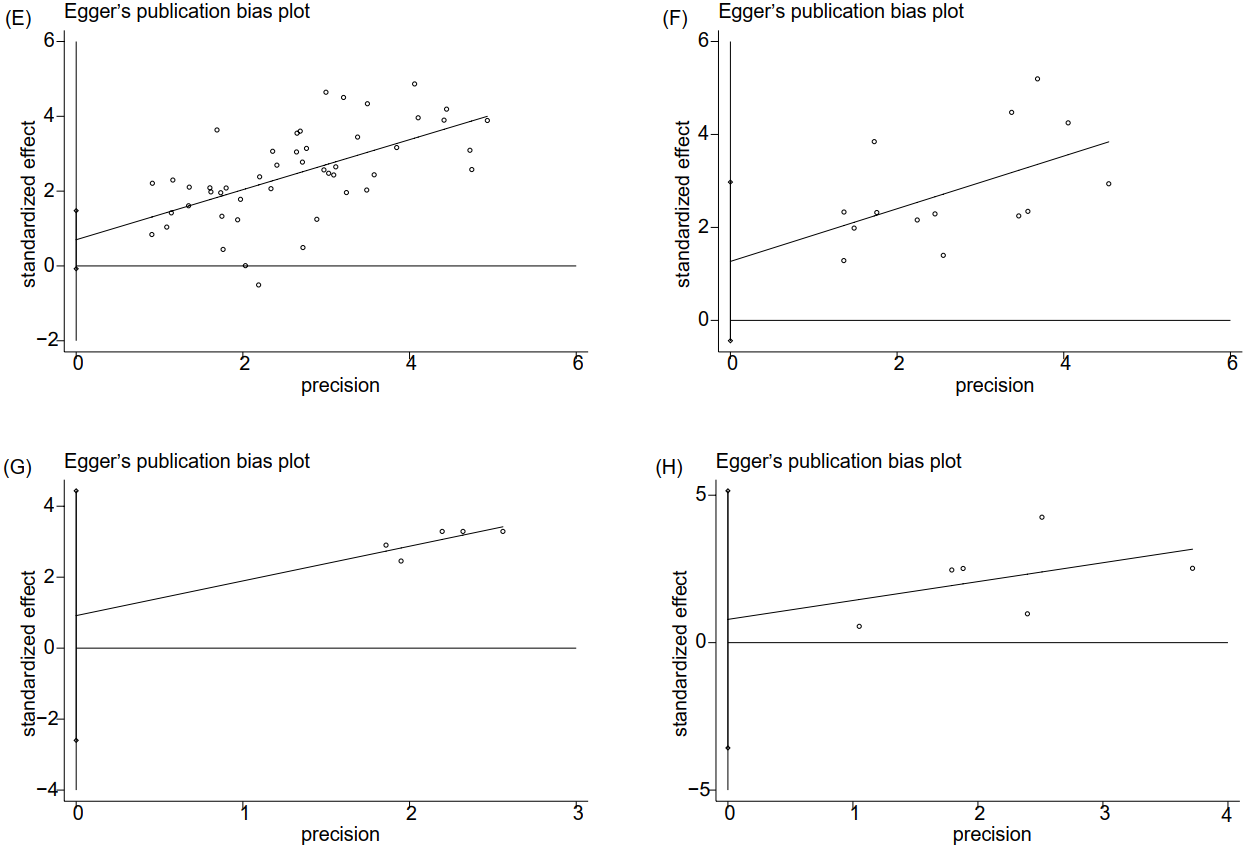


**Supplementary Figure S4.** Begg’s and Eggers’ funnel plots for publication bias of prognostic value of POU5F1. Begg’s funnel plots: (A) OS; (B) DFS; (C) DSS; (D) RFS. Eggers’ funnel plots: (E) OS; (F) DFS; (G) DSS; (H) RFS. logHR, logarithm of hazard ratios; s.e., standard error.
