# Supplementary material for: Diagnostic significance and carcinogenic mechanism of pan‐cancer gene POU5F1 in liver hepatocellular carcinoma
Source: Cancer Med. 2020 Sep 26;9(23):8782–800. doi: 10.1002/cam4.3486 (PMC7724499; doi:10.1002/cam4.3486)
Supplement: Supplementary file 5 — Fig S5 [file CAM4-9-8782-s005.docx]

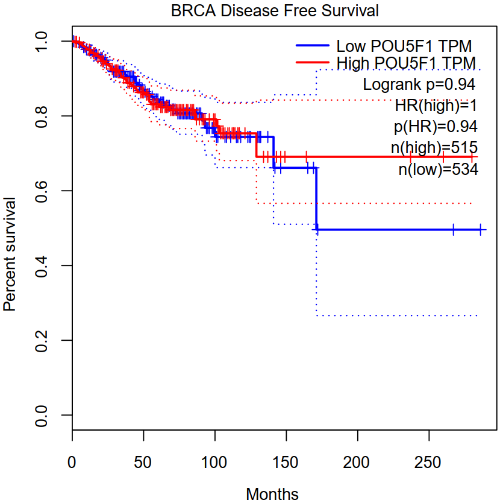

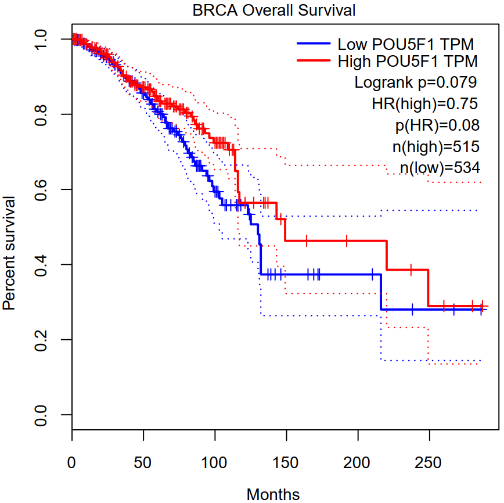

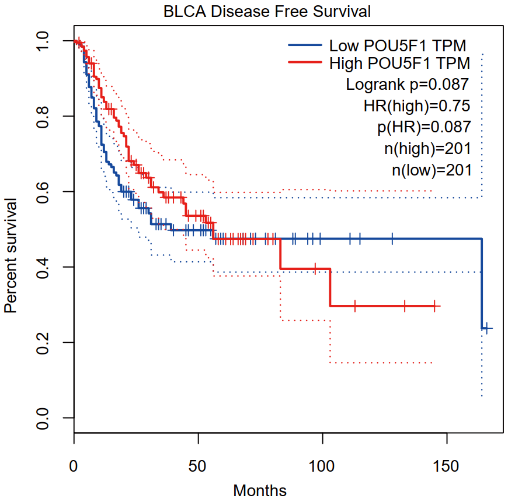

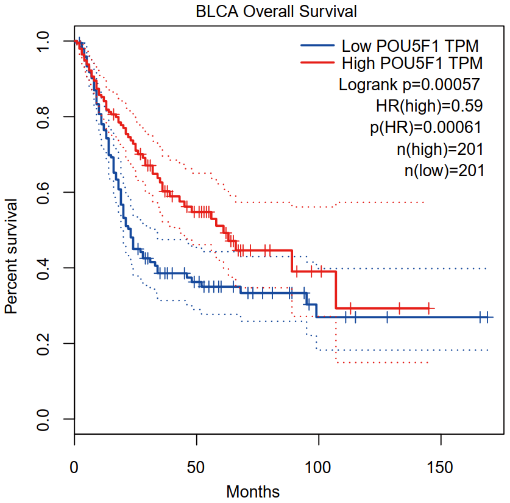


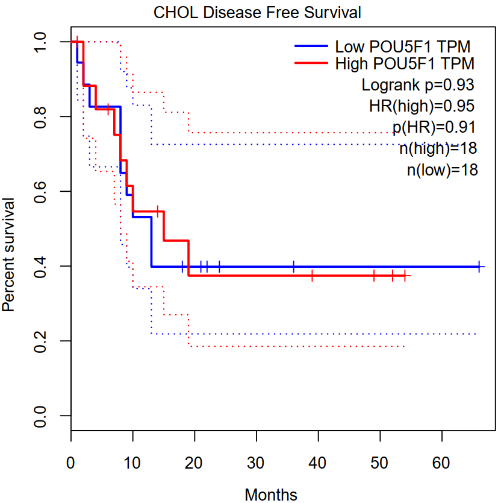

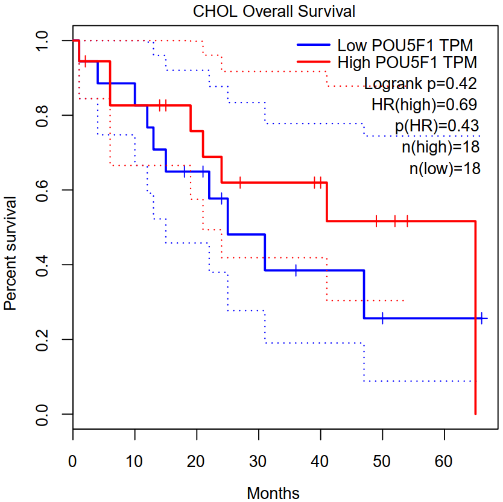


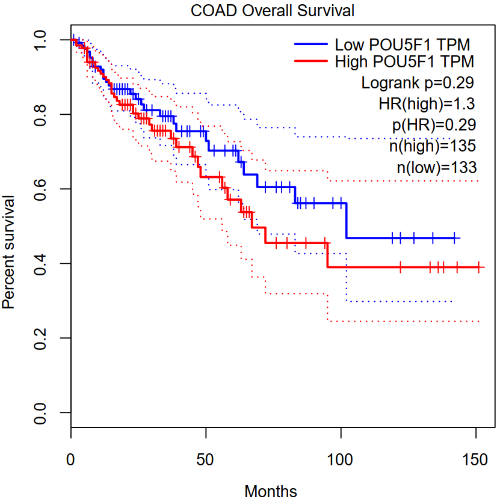

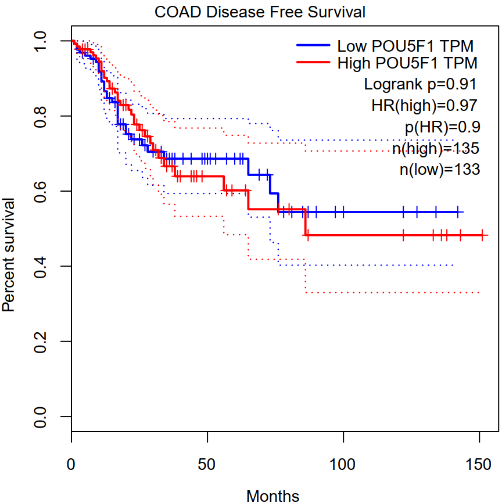


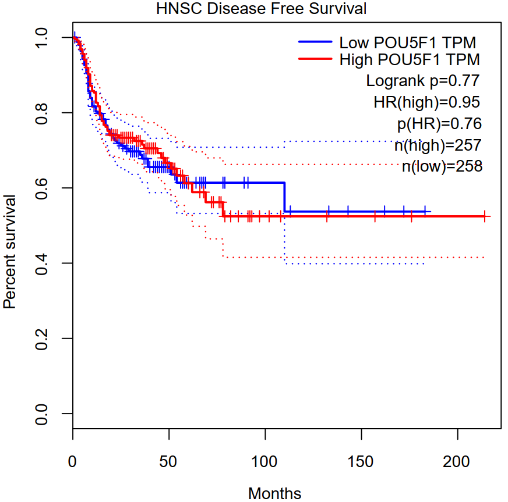

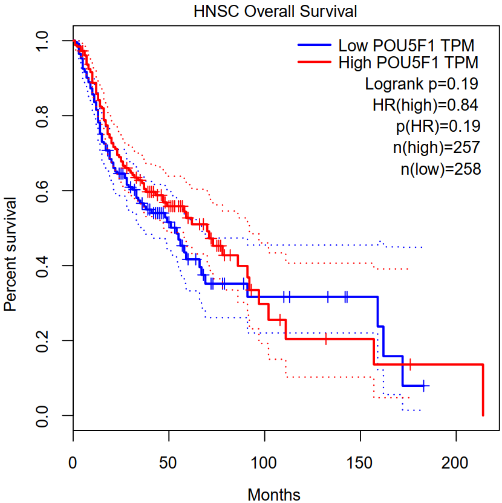

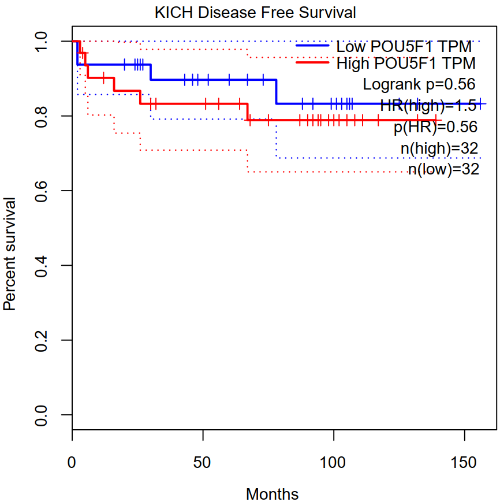

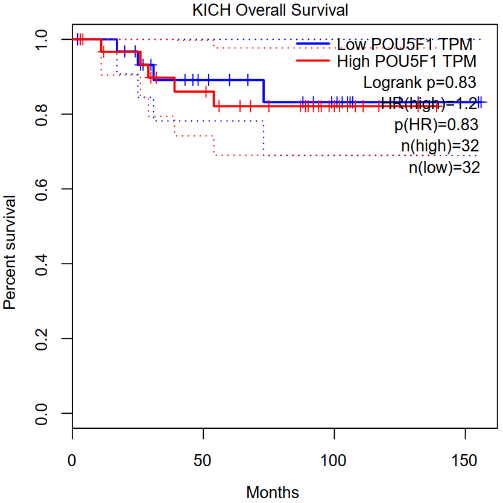


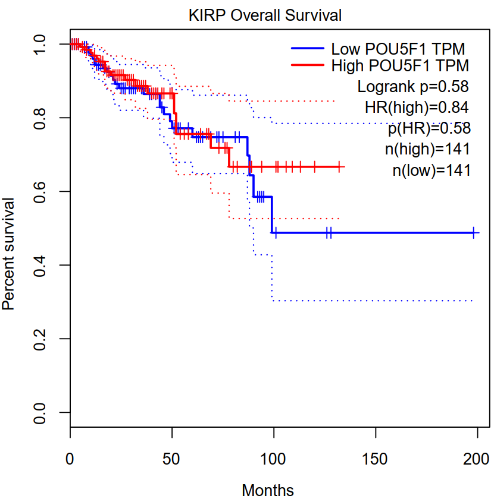

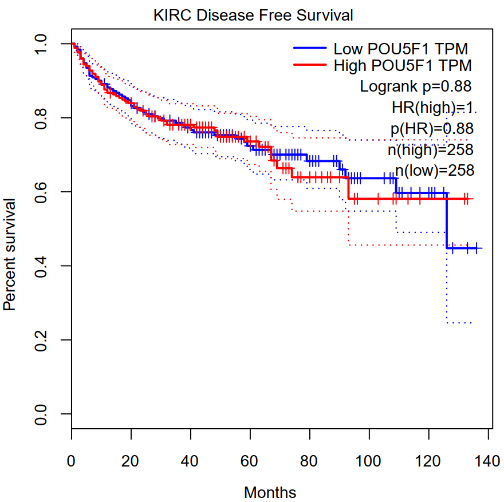

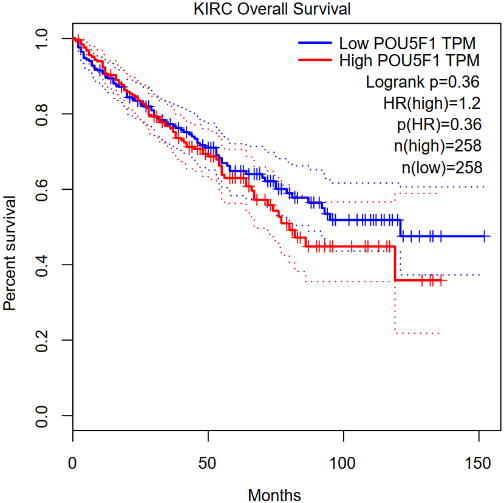

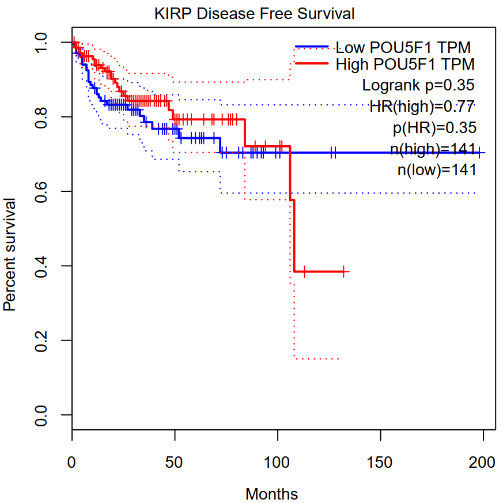


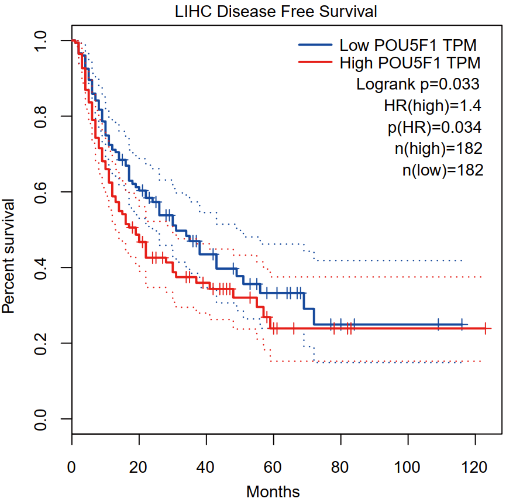

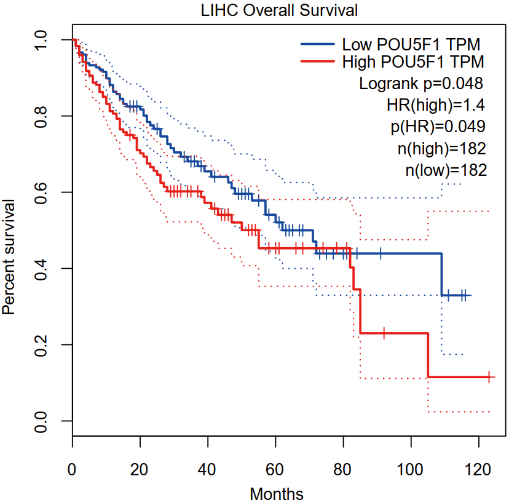


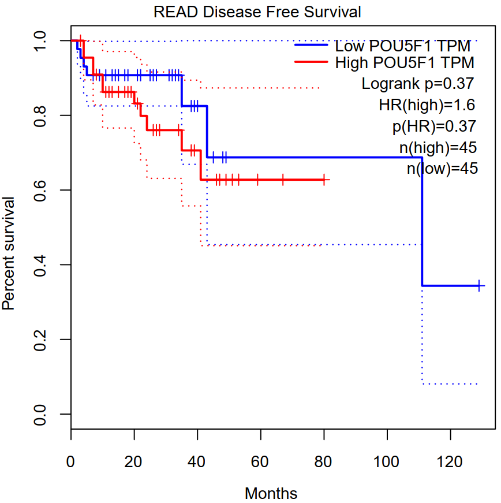

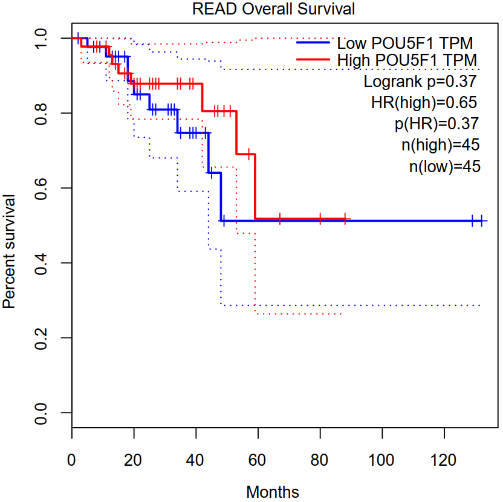


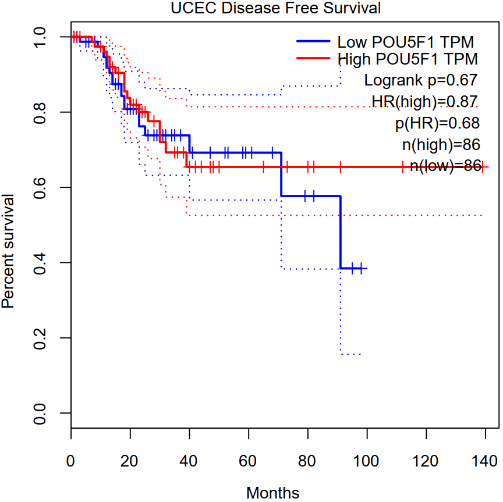

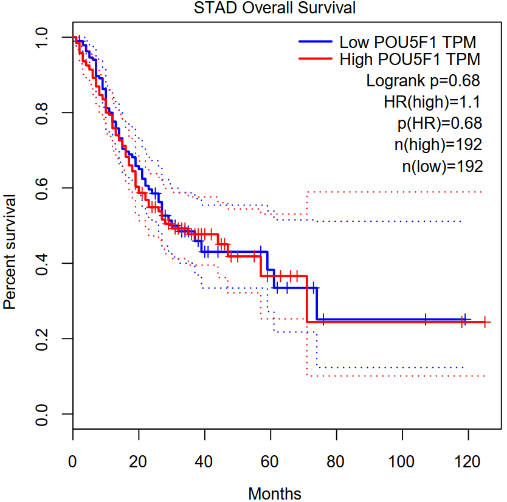

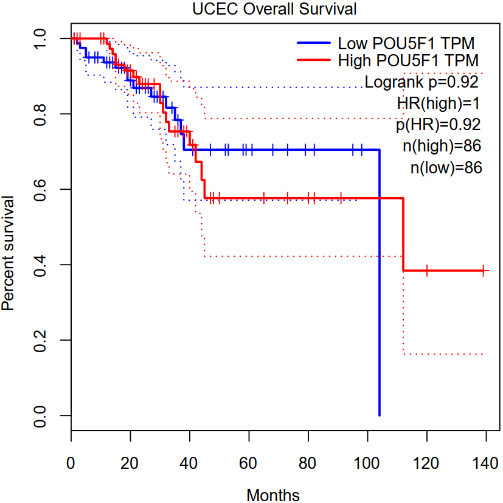

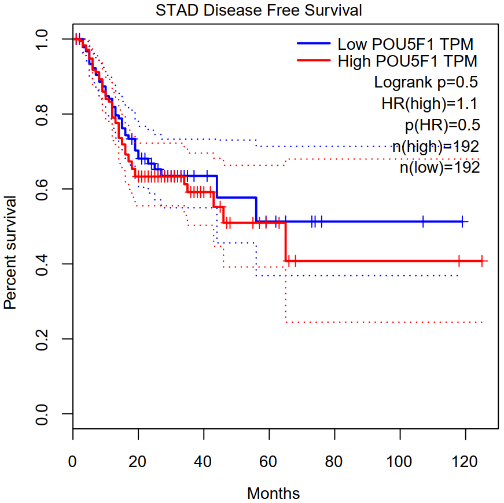


**Supplementary Figure S5.** Kaplan–Meier survival analysis of 12 types cancers based on TCGA. BLCA, bladder urothelial carcinoma ; BRCA, breast invasive carcinoma; CHOL, cholangiocarcinoma ; COAD, colon adenocarcinoma, ; HNSC, head and neck squamous cell carcinoma; KICH, kidney chromophobe; KIRC, kidney renal clear cell carcinoma, ; KIRP, kidney renal papillary cell carcinoma; LIHC, liver hepatocellular carcinoma; READ, rectum adenocarcinoma; STAD, stomach adenocarcinoma; UCEC , uterine corpus endometrial carcinoma.
