# Supplementary material for: Diagnostic significance and carcinogenic mechanism of pan‐cancer gene POU5F1 in liver hepatocellular carcinoma
Source: Cancer Med. 2020 Sep 26;9(23):8782–800. doi: 10.1002/cam4.3486 (PMC7724499; doi:10.1002/cam4.3486)
Supplement: Supplementary file 6 — Table S1 [file CAM4-9-8782-s006.docx]

**Supplementary Table S1** Primer sequence and Tm for qPCR.

| **Gene** | **Primer sequence（5’→3’）** | **Tm（℃）** | **Product length（bp）** |
| --- | --- | --- | --- |
| POU5F1 | F: CGAAAGAGAAAGCGAACCAG | 60 | 157 |
|  | R: GCCGGTTACAGAACCACACT |  |  |
| GAPDH | F: GAAGGTGAAGGTCGGAGTC | 60 | 226 |
|  | R: GAAGATGGTGATGGGATTTC |  |  |
