# Supplementary material for: Diagnostic significance and carcinogenic mechanism of pan‐cancer gene POU5F1 in liver hepatocellular carcinoma
Source: Cancer Med. 2020 Sep 26;9(23):8782–800. doi: 10.1002/cam4.3486 (PMC7724499; doi:10.1002/cam4.3486)
Supplement: Supplementary file 7 — Table S2 [file CAM4-9-8782-s007.docx]

**Supplementary Table S2** Baseline characteristics of included studies

| **Reference** | **Year** | **Country** | **No. of  patients** | **Tumor type** | **Outcome** | **Age, year** | | **Gender** | | **Follow-up, month** | | **Detection  method** | **Cut-off  value** | **Hazard ratio [HR]   [Tierney method]** | **NOS** |
| --- | --- | --- | --- | --- | --- | --- | --- | --- | --- | --- | --- | --- | --- | --- | --- |
|  |  |  |  |  |  | **Median** | **Range** | **M** | **F** | **Median** | **Range** |  |  |  |  |
| Li et al. [10] | 2012 | China | 50 | Esophageal squamous cell carcinoma | OS | 62 | 47-72 | 37 | 13 | 34.5 | NR | IHC | IHC ≥ 5% | Reported in text | 6 |
| Cao et al. [11] | 2013 | China | 49 | Hepatocellular carcinoma | OS/DFS | 49 | NR | 37 | 12 | 12.7 | 1.6-34.2 | IHC | IHC score≥ 2 | Kaplan-Meier curves | 6 |
| Chang et al. [12] | 2008 | China | 57 | Bladder transitional cell carcinoma | OS | NR | 40-82 | 24 | 23 | NR | 14-67 | IHC | NR | Kaplan-Meier curves | 8 |
| Chen et al. [13] | 2008 | China | 78 | Non-small-cell lung carcinoma | OS | NR | NR | NR | NR | NR | NR | IHC | NR | Kaplan-Meier curves | 7 |
| Chiou et al. [14] | 2008 | China | 52 | Oral squamous cell carcinoma | OS | NR | NR | NR | NR | NR | NR | IHC | IHC ≥ 5% | Kaplan-Meier curves | 8 |
| He et al. [15] | 2012 | China | 153 | Esophageal squamous cell carcinoma | OS | NR | 33-73 | 93 | 60 | 61 | 1-139 | IHC | IHC score≥1 | Reported in text | 8 |
| Ge et al. [16] | 2010 | China | 85 | Hypopharyngeal squamous cell carcinoma | OS | 60 | 37-82 | 84 | 1 | 17 | 0.2-74 | IHC | IHC score≥ 4 | Kaplan-Meier curves | 8 |
| Qian et al. [17] | 2012 | China | 130 | Hepatocellular carcinoma | OS/DFS | 50 | 22-78 | 116 | 14 | 45 | 1-92 | IHC | IHC score≥ 2 | Formula | 8 |
| Huang et al. [18] | 2011 | China | 136 | Hepatocellular carcinoma | OS/DFS | 50 | NR | 122 | 14 | 20 | 1-83 | RT-PCR | NR | Reported in text | 8 |
| Cortes et al. [27] | 2012 | Switzerland | 64 | Lung adenocarcinoma | DFS | 62 | 34-86 | 34 | 30 | 13 | 1月29日 | RT-PCR | NR | Reported in text | 5 |
| Ravindran et al. [28] | 2015 | India | 60 | Oral squamous cell carcinoma | OS/DFS | NR | 45-70 | 34 | 26 | NR | 14-48 | IHC | IHC ≥ 16% | Reported in text | 7 |
| Chang et al. [29] | 2015 | China | 191 | Hepatocellular carcinoma | OS/RFS | 55 | 21-80 | 146 | 45 | NR | 1.2-106 | RT-PCR | T/PT ≥ 2-fold | Kaplan-Meier curves | 7 |
| Chiou et al. [30] | 2010 | China | 118 | Lung adenocarcinoma | OS | 58 | 45-79 | NR | NR | NR | NR | IHC | NR | Kaplan-Meier curves | 6 |
| Comisso et al. [31] | 2017 | Italy | 23 | High-grade serous ovarian cancer | OS | NR | NR | – | 23 | NR | NR | RT-PCR | NR | Kaplan-Meier curves | 7 |
| Dong et al. [32] | 2012 | China | 152 | Hepatocellular carcinoma | OS | 50 | NR | 133 | 19 | NR | NR | IHC | IHC score≥ 4 | Reported in text | 7 |
| Gwak et al. [33] | 2017 | Korea | 319 | Breast cancer | DFS | 50.9 | 26-87 | – | 319 | 63.5 | 0.5-128 | IHC | IHC ≥ 10% | Reported in text | 7 |
| Hu et al. [34] | 2017 | China | 143 | Right-sided colon cancer | OS | NR | 21-93 | 66 | 77 | 68.1 | NR | IHC | IHC score≥ 4 | Formula | 7 |
| Huang et al. [35] | 2012 | Japan | 78 | Bladder malignancy | RFS | NR | 22–88 | 64 | 14 | NR | 36-72 | IHC | IHC ≥ 1% | Kaplan-Meier curves | 9 |
| Javanbakht et al. [36] | 2017 | Iran | 40 | Gastric cancer | OS | NR | 37-85 | 16 | 24 | NR | NR | IHC | NR | Reported in text | 8 |
| Jen et al. [37] | 2017 | China | 124 | Lung cancer | OS | 65 | NR | NR | NR | NR | NR | RT-PCR | NR | Reported in text | 7 |
| Jiang et al. [38] | 2016 | China | 412 | Gastric cancer | OS | 62 | 30-85 | 284 | 128 | NR | NR | IHC | IHC score≥ 4 | Reported in text | 7 |
| Kaneko et al. [39] | 2015 | Japan | 36 | Neuroblastomas | OS | 1.5 | NR | NR | NR | NR | NR | RT-PCR | NR | Reported in text | 5 |
| Kim et al. [40] | 2015 | Korea | 161 | Cervical cancer | OS/DFS | NR | 19-83 | – | 161 | NR | 1-179 | IHC | HSCORE> 200 | Reported in text | 9 |
| Kim et al. [41] | 2012 | Korea | 119 | Papillary renal cell carcinoma | DSS | 54 | 11-75 | 86 | 33 | NR | 0.6-158 | IHC | IHC > 12.5% | Reported in text | 9 |
| Kong et al. [42] | 2014 | China | 158 | Gastric cancer | OS | 60 | NR | 104 | 54 | 38 | NR | IHC | IHC score≥ 1 | Reported in text | 7 |
| Kosaka et al. [43] | 2016 | Japan | 205 | Prostate cancer | RFS | 67.4 | 49-75 | 205 | – | 79.2 | NR | IHC | IHC score≥ 1 | Reported in text | 7 |
| Li et al. [44] | 2017 | China | 67 | Esophageal cancer | OS | 64 | 45-73 | 52 | 15 | 65 | 9-106 | IHC | IHC ≥ 10% | Kaplan-Meier curves | 8 |
| Li et al. [45] | 2015 | China | 69 | Gastric cancer | OS/DFS | 55 | 28-78 | 51 | 18 | 35 | 6-50 | IHC | IHC score≥ 5 | Reported in text | 7 |
| Li et al. [46] | 2012 | China | 44 | Non-small-cell lung cancer | OS | NR | 37-78 | 32 | 12 | NR | NR | IHC | IHC ≥ 10% | Kaplan-Meier curves | 7 |
| Li et al. [47] | 2013 | China | 102 | Non-small-cell lung cancer | OS | 60 | NR | 59 | 43 | NR | NR | IHC | IHC score≥ 4 | Kaplan-Meier curves | 7 |
| Liu et al. [48] | 2012 | China | 320 | Breast cancer | DSS | 35 | NR | – | 320 | NR | NR | IHC | IHC ≥ 1% | Formula | 6 |
| Liu et al. [49] | 2011 | China | 126 | Breast cancer | DSS | 35 | NR | – | 126 | NR | NR | IHC | IHC ≥ 1% | Formula | 7 |
| Liu et al. [50] | 2014 | China | 90 | Breast cancer | OS/DFS | 50 | NR | – | 90 | 86.6 | 5-149 | IHC | IHC ≥ 10% | Reported in text | 8 |
| Lu et al. [51] | 2013 | China | 43 | Pancreatic cancer | OS | NR | NR | NR | NR | NR | NR | IHC | IHC score ≥ 5 | Reported in text | 6 |
| Luo et al. [52] | 2013 | China | 122 | Nasopharyngeal carcinoma | OS | 47.6 | 15-73 | 92 | 30 | NR | 8-92 | IHC | IHC score ≥ 6 | Reported in text | 9 |
| Matsuoka et al. [53] | 2012 | Japan | 290 | Gastric carcinoma | OS | NR | NR | NR | NR | NR | NR | IHC | IHC score ≥ 5 | Reported in text | 7 |
| Miyoshi et al. [54] | 2018 | Japan | 95 | Colorectal cancer | OS/DFS | 66 | NR | 41 | 54 | 53.9 | NR | RT-PCR | 0.5 | Reported in text | 7 |
| Sawant et al. [55] | 2016 | India | 87 | Oral squamous cell carcinoma | OS/DFS | 50 | NR | 75 | 12 | 25 | NR | IHC | IHC score≥ 3 | Reported in text | 7 |
| Tang et al. [56] | 2015 | China | 133 | Lung cancer | OS | 60 | NR | 93 | 40 | NR | NR | IHC | IHC ≥ 50% | Reported in text | 7 |
| Wang et al. [57] | 2014 | China | 126 | Breast cancer | OS | 35 | NR | – | 126 | NR | NR | IHC | IHC ≥ 1% | Kaplan-Meier curves | 6 |
| Wang et al. [58] | 2018 | China | 53 | Hepatocellular carcinoma | OS/RFS | 50 | 36‑67 | 39 | 14 | 39 | 5‑60 | IHC | IHC score> 4 | Reported in text | 8 |
| Wang et al. [59] | 2018 | China | 151 | Colon cancer | OS/DFS | 60 | NR | 62 | 89 | NR | 3-60 | IHC | IHC score> 10 | Reported in text | 9 |
| Xiang et al. [60] | 2018 | China | 152 | Acute myeloid leukemia | OS/DFS | 37 | NR | 89 | 63 | 17 | 2-36 | RT-PCR | 1.101 | Reported in text | 6 |
| Xin et al. [61] | 2013 | China | 55 | Lung adenocarcinoma | DSS | NR | NR | 30 | 25 | NR | NR | IHC | NR | Kaplan-Meier curves | 7 |
| Xing et al. [62] | 2010 | China | 52 | Rectal Adenocarcinoma | OS/RFS | 63 | 17-88 | 28 | 24 | 34 | 2-42 | IHC | IHC ≥ 5% | Formula | 7 |
| Yang et al. [63] | 2014 | China | 630 | Cervical cancer | DSS | NR | 35-81 | – | 630 | NR | 2-117 | IHC | IHC score≥ 3 | Formula | 8 |
| Yin et al. [64] | 2015 | China | 87 | Acute myeloid leukemia | OS | 55 | 10-87 | 47 | 40 | NR | NR | RT-PCR | 0.56 | Kaplan-Meier curves | 6 |
| Yin et al. [65] | 2013 | China | 57 | Hepatocellular carcinoma | OS/RFS | 49 | 33-67 | 48 | 9 | 22 | 5-58 | RT-PCR | X-title >4.0 | Reported in text | 7 |
| Yin et al. [66] | 2012 | China | 228 | Hepatocellular carcinoma | OS | 51.4 | 35-80 | 194 | 34 | 60 | 1-83 | IHC | IHC score≥ 1 | Formula | 7 |
| You et al. [67] | 2018 | China | 153 | Rectal cancer | OS | NR | NR | 83 | 70 | 62.4 | NR | IF | HSCORE≥ 0.7 | Reported in text | 7 |
| Zhang et al. [68] | 2018 | China | 127 | Triple‑negative breast cancer | OS | NR | NR | – | 127 | NR | NR | IF | HSCORE≥ 0.7 | Reported in text | 6 |
| Zhang et al. [69] | 2010 | China | 107 | Lung adenocarcinoma | OS | 57 | 22-81 | 56 | 51 | NR | NR | IF | HSCORE≥ 0.7 | Reported in text | 7 |
| Zhao et al. [70] | 2016 | China | 86 | Hepatocellular carcinoma | OS/DFS | 60 | NR | 67 | 19 | 31.5 | 3-72 | IHC | IHC score≥ 4 | Reported in text | 8 |
| Zhao et al. [71] | 2018 | China | 95 | Esophageal squamous cell carcinoma | OS | 65 | NR | 78 | 17 | 27 | NR | IHC | IHC score≥ 2 | Kaplan-Meier curves | 7 |
| Zhou et al. [72] | 2015 | China | 158 | Colorectal cancer | OS | 64 | 36-76 | 96 | 62 | 59 | NR | IHC | NR | Kaplan-Meier curves | 7 |
| Zhou et al. [73] | 2016 | China | 195 | Bladder cancer | OS | NR | NR | 153 | 42 | NR | NR | IHC | IHC score≥ 2 | Kaplan-Meier curves | 5 |
| Zou et al. [74] | 2013 | China | 108 | Gallbladder adenocarcinoma | OS | NR | NR | 31 | 77 | NR | NR | IHC | IHC score≥ 3 | Reported in text | 7 |

NR: not reported; M: male; F: female; NOS: Newcastle-Ottawa Scale; OS: overall survival; DFS: disease free survival; DSS: disease specific survival; RFS: recurrence free survival; IHC: immunohistochemistry; RT-PCR: reverse transcription polymerase chain reaction; IF: immunofluorescence.
