# Supplementary material for: Diagnostic significance and carcinogenic mechanism of pan‐cancer gene POU5F1 in liver hepatocellular carcinoma
Source: Cancer Med. 2020 Sep 26;9(23):8782–800. doi: 10.1002/cam4.3486 (PMC7724499; doi:10.1002/cam4.3486)
Supplement: Supplementary file 8 — Table S3 [file CAM4-9-8782-s008.docx]

**Supplementary Table S3** Methodological quality assessment of observational studies based on the Newcastle-Ottawa Scale (NOS)

| **Reference** | **Year** | Selection | | | |  | Comparability | |  | Outcome | | | Total score |
| --- | --- | --- | --- | --- | --- | --- | --- | --- | --- | --- | --- | --- | --- |
|  |  | Representativeness of the exposed cohort | Selection of the non exposed cohort | Ascertainment of exposure | Demonstration that outcome of interest was not present at start of study |  | Study design (cohort study) | Study controls for any additional factor |  | Assessment of outcome | Follow-up long enough (≥5 years) | Adequacy of follow up of cohorts (>80%) |  |
| Li et al. [10] | 2012 |  |  | ★ | ★ |  | ★ | ★ |  | ★ | ★ |  | 6 |
| Cao et al. [11] | 2013 |  | ★ | ★ | ★ |  | ★ | ★ |  | ★ |  |  | 6 |
| Chang et al. [12] | 2008 | ★ | ★ |  | ★ |  | ★ | ★ |  | ★ | ★ | ★ | 8 |
| Chen et al. [13] | 2008 | ★ | ★ |  | ★ |  | ★ | ★ |  | ★ |  | ★ | 7 |
| Chiou et al. [14] | 2008 | ★ | ★ | ★ | ★ |  | ★ | ★ |  | ★ |  | ★ | 8 |
| He et al. [15] | 2012 | ★ | ★ | ★ | ★ |  | ★ | ★ |  | ★ | ★ |  | 8 |
| Ge et al. [16] | 2010 | ★ | ★ | ★ | ★ |  | ★ | ★ |  |  | ★ | ★ | 8 |
| Qian et al. [17] | 2012 | ★ | ★ | ★ | ★ |  | ★ |  |  | ★ | ★ | ★ | 8 |
| Huang et al. [18] | 2011 | ★ | ★ |  | ★ |  | ★ | ★ |  | ★ | ★ | ★ | 8 |
| Cortes et al. [27] | 2012 | ★ |  |  | ★ |  | ★ |  |  | ★ |  | ★ | 5 |
| Ravindran et al. [28] | 2015 | ★ |  | ★ | ★ |  | ★ | ★ |  | ★ |  | ★ | 7 |
| Chang et al. [29] | 2015 | ★ |  | ★ | ★ |  | ★ | ★ |  |  | ★ | ★ | 7 |
| Chiou et al. [30] | 2010 | ★ | ★ |  | ★ |  | ★ |  |  | ★ |  | ★ | 6 |
| Comisso et al. [31] | 2017 | ★ | ★ |  | ★ |  | ★ | ★ |  | ★ |  | ★ | 7 |
| Dong et al. [32] | 2012 |  | ★ | ★ | ★ |  | ★ | ★ |  | ★ |  | ★ | 7 |
| Gwak et al. [33] | 2017 | ★ |  | ★ | ★ |  | ★ |  |  | ★ | ★ | ★ | 7 |
| Hu et al. [34] | 2017 |  | ★ | ★ | ★ |  | ★ | ★ |  |  | ★ | ★ | 7 |
| Huang et al. [35] | 2012 | ★ | ★ | ★ | ★ |  | ★ | ★ |  | ★ | ★ | ★ | 9 |
| Javanbakht et al. [36] | 2017 | ★ | ★ | ★ | ★ |  | ★ | ★ |  | ★ |  | ★ | 8 |
| Jen et al. [37] | 2017 | ★ | ★ | ★ | ★ |  | ★ |  |  | ★ |  | ★ | 7 |
| Jiang et al. [38] | 2016 | ★ | ★ | ★ | ★ |  | ★ |  |  | ★ |  | ★ | 7 |
| Kaneko et al. [39] | 2015 |  |  |  | ★ |  | ★ | ★ |  | ★ |  | ★ | 5 |
| Kim et al. [40] | 2015 | ★ | ★ | ★ | ★ |  | ★ | ★ |  | ★ | ★ | ★ | 9 |
| Kim et al. [41] | 2012 | ★ | ★ | ★ | ★ |  | ★ | ★ |  | ★ | ★ | ★ | 9 |
| Kong et al. [42] | 2014 | ★ |  | ★ | ★ |  | ★ | ★ |  | ★ | ★ |  | 7 |
| Kosaka et al. [43] | 2016 |  | ★ | ★ | ★ |  | ★ | ★ |  |  | ★ | ★ | 7 |
| Li et al. [44] | 2017 | ★ | ★ | ★ | ★ |  | ★ | ★ |  |  | ★ | ★ | 8 |
| Li et al. [45] | 2015 | ★ |  | ★ | ★ |  | ★ | ★ |  |  | ★ | ★ | 7 |
| Li et al. [46] | 2012 | ★ | ★ | ★ | ★ |  | ★ | ★ |  |  |  | ★ | 7 |
| Li et al. [47] | 2013 |  | ★ | ★ | ★ |  | ★ | ★ |  | ★ |  | ★ | 7 |
| Liu et al. [48] | 2012 |  | ★ | ★ | ★ |  | ★ | ★ |  | ★ |  |  | 6 |
| Liu et al. [49] | 2011 | ★ | ★ | ★ | ★ |  | ★ | ★ |  |  |  | ★ | 7 |
| Liu et al. [50] | 2014 | ★ |  | ★ | ★ |  | ★ | ★ |  | ★ | ★ | ★ | 8 |
| Lu et al. [51] | 2013 | ★ |  | ★ | ★ |  | ★ | ★ |  | ★ |  |  | 6 |
| Luo et al. [52] | 2013 | ★ | ★ | ★ | ★ |  | ★ | ★ |  | ★ | ★ | ★ | 9 |
| Matsuoka et al. [53] | 2012 | ★ | ★ | ★ | ★ |  | ★ | ★ |  | ★ |  |  | 7 |
| Miyoshi et al. [54] | 2018 |  | ★ |  | ★ |  | ★ | ★ |  | ★ | ★ | ★ | 7 |
| Sawant et al. [55] | 2016 | ★ |  | ★ | ★ |  | ★ | ★ |  | ★ |  | ★ | 7 |
| Tang et al. [56] | 2015 | ★ | ★ | ★ | ★ |  | ★ | ★ |  | ★ |  |  | 7 |
| Wang et al. [57] | 2014 | ★ | ★ |  | ★ |  | ★ | ★ |  | ★ |  |  | 6 |
| Wang et al. [58] | 2018 | ★ | ★ | ★ | ★ |  | ★ | ★ |  |  | ★ | ★ | 8 |
| Wang et al. [59] | 2018 | ★ | ★ | ★ | ★ |  | ★ | ★ |  | ★ | ★ | ★ | 9 |
| Xiang et al. [60] | 2018 |  | ★ |  | ★ |  | ★ | ★ |  | ★ |  | ★ | 6 |
| Xin et al. [61] | 2013 | ★ | ★ | ★ | ★ |  | ★ | ★ |  | ★ |  |  | 7 |
| Xing et al. [62] | 2010 | ★ |  | ★ | ★ |  | ★ | ★ |  | ★ |  | ★ | 7 |
| Yang et al. [63] | 2014 | ★ | ★ | ★ | ★ |  | ★ | ★ |  |  | ★ | ★ | 8 |
| Yin et al. [64] | 2015 |  |  | ★ | ★ |  | ★ | ★ |  | ★ |  | ★ | 6 |
| Yin et al. [65] | 2013 | ★ | ★ |  | ★ |  | ★ | ★ |  |  | ★ | ★ | 7 |
| Yin et al. [66] | 2012 | ★ | ★ |  | ★ |  | ★ | ★ |  | ★ | ★ |  | 7 |
| You et al. [67] | 2018 |  | ★ | ★ | ★ |  | ★ |  |  | ★ | ★ | ★ | 7 |
| Zhang et al. [68] | 2018 |  |  | ★ | ★ |  | ★ | ★ |  |  | ★ | ★ | 6 |
| Zhang et al. [69] | 2010 | ★ | ★ | ★ | ★ |  | ★ |  |  | ★ |  | ★ | 7 |
| Zhao et al. [70] | 2016 | ★ | ★ | ★ | ★ |  | ★ |  |  | ★ | ★ | ★ | 8 |
| Zhao et al. [71] | 2018 | ★ | ★ | ★ | ★ |  | ★ | ★ |  | ★ |  |  | 7 |
| Zhou et al. [72] | 2015 | ★ |  | ★ | ★ |  | ★ | ★ |  | ★ | ★ |  | 7 |
| Zhou et al. [73] | 2016 |  |  | ★ | ★ |  | ★ | ★ |  |  |  | ★ | 5 |
| Zou et al. [74] | 2013 | ★ |  | ★ | ★ |  | ★ | ★ |  | ★ |  | ★ | 7 |
