# Supplementary material for: Diagnostic significance and carcinogenic mechanism of pan‐cancer gene POU5F1 in liver hepatocellular carcinoma
Source: Cancer Med. 2020 Sep 26;9(23):8782–800. doi: 10.1002/cam4.3486 (PMC7724499; doi:10.1002/cam4.3486)
Supplement: Supplementary file 9 — Table S4 [file CAM4-9-8782-s009.docx]

**Supplementary Table S4** Subgroup analyses of pooled HRs for DFS

| **Categories** | | **No. of  studies** | **No. of  patients** | **Pooled HR**  **(95% CI)** | **Significant  *z*** | ***P*-value** | **Heterogeneity  *I*^2^ (%)** | ***P*-value** | **Model** |
| --- | --- | --- | --- | --- | --- | --- | --- | --- | --- |
|  |  |  |  |  |  |  |  |  |  |
| **[1]** | **DFS** | 14 | 1649 | 2.66 (2.22-3.19) | 10.54 | 0.000 | 20.7 | 0.229 | Fixed |
| **[2]** | **Cancer type** |  |  |  |  |  |  |  |  |
| 1) | Head and neck cancer | 2 | 147 | 7.61 (3.12-18.56) | 4.46 | 0.000 | 0.0 | 0.579 | Fixed |
| 2) | Breast cancer | 2 | 409 | 3.54 (2.27-5.51) | 5.60 | 0.000 | 0.0 | 0.329 | Fixed |
| 3) | Hepatocellular cancer | 4 | 401 | 2.39 (1.74-3.28) | 5.36 | 0.000 | 15.3 | 0.315 | Fixed |
| 4) | Colorectal cancer | 2 | 246 | 2.03 (1.38-2.99) | 3.59 | 0.000 | 0.0 | 0.523 | Fixed |
| 5) | Other cancers | 4 | 446 | 2.68 (1.85-3.87) | 5.24 | 0.000 | 0.0 | 0.586 | Fixed |
| **[3]** | **Analysis type** |  |  |  |  |  |  |  |  |
| 1) | Multivariate | 12 | 1470 | 2.56 (2.11-3.10) | 9.53 | 0.000 | 25.9 | 0.190 | Fixed |
| 2) | Univariate | 2 | 179 | 3.57 (2.08-6.12) | 4.63 | 0.000 | 0.0 | 0.629 | Fixed |
| **[4]** | **Sample size** |  |  |  |  |  |  |  |  |
| 1) | ≥110 | 6 | 1049 | 2.29 (1.78-2.95) | 6.47 | 0.000 | 0.0 | 0.748 | Fixed |
| 2) | <110 | 8 | 600 | 3.12 (2.40-4.06) | 8.48 | 0.000 | 35.9 | 0.142 | Fixed |
| **[5]** | **Detection method** |  |  |  |  |  |  |  |  |
| 1) | IHC | 10 | 1202 | 2.73 (2.19-3.40) | 8.97 | 0.000 | 38.6 | 0.101 | Fixed |
| 2) | RT-PCR | 4 | 447 | 2.50 (1.81-3.46) | 5.53 | 0.000 | 0.0 | 0.673 | Fixed |
